# The Mitochondrial Calcium Uniporter Interacts with Subunit c of the ATP Synthase of Trypanosomes and Humans

**DOI:** 10.1128/mBio.00268-20

**Published:** 2020-03-17

**Authors:** Guozhong Huang, Roberto Docampo

**Affiliations:** a Center for Tropical and Emerging Global Diseases, University of Georgia, Athens, Georgia, USA; b Department of Cellular Biology, University of Georgia, Athens, Georgia, USA; Washington University School of Medicine

**Keywords:** ATP synthase, c ring, *Trypanosoma*, mitochondrial calcium uniporter

## Abstract

Mitochondrial Ca^2+^ transport mediated by the uniporter complex (MCUC) plays a key role in the regulation of cell bioenergetics in both trypanosomes and mammals. Here we report that Trypanosoma brucei MCU (TbMCU) subunits interact with subunit c of the mitochondrial ATP synthase (ATPc), as determined by coimmunoprecipitation and split-ubiquitin membrane-based yeast two-hybrid (MYTH) assays. Mutagenesis analysis in combination with MYTH assays suggested that transmembrane helices (TMHs) are determinants of this specific interaction. *In situ* tagging, followed by immunoprecipitation and immunofluorescence microscopy, revealed that T. brucei ATPc (TbATPc) coimmunoprecipitates with TbMCUC subunits and colocalizes with them to the mitochondria. Blue native PAGE and immunodetection analyses indicated that the TbMCUC is present together with the ATP synthase in a large protein complex with a molecular weight of approximately 900 kDa. Ablation of the *TbMCUC* subunits by RNA interference (RNAi) significantly increased the AMP/ATP ratio, revealing the downregulation of ATP production in the cells. Interestingly, the direct physical MCU-ATPc interaction is conserved in Trypanosoma cruzi and human cells. Specific interaction between human MCU (HsMCU) and human ATPc (HsATPc) was confirmed *in vitro* by mutagenesis and MYTH assays and *in vivo* by coimmunoprecipitation. In summary, our study has identified that MCU complex physically interacts with mitochondrial ATP synthase, possibly forming an MCUC-ATP megacomplex that couples ADP and P_i_ transport with ATP synthesis, a process that is stimulated by Ca^2+^ in trypanosomes and human cells.

## Abstract (precis)

The mitochondrial calcium uniporter (MCU) is essential for the regulation of oxidative phosphorylation in mammalian cells, and we have shown that in Trypanosoma brucei, the etiologic agent of sleeping sickness, this channel is essential for its survival and infectivity. Here we reveal that that Trypanosoma brucei MCU subunits interact with subunit c of the mitochondrial ATP synthase (ATPc). Interestingly, the direct physical MCU-ATPc interaction is conserved in T. cruzi and human cells.

## INTRODUCTION

The Trypanosoma brucei group of parasites causes nagana in cattle and African trypanosomiasis, or sleeping sickness, in humans. Two of the best-studied life cycle stages of T. brucei are the procyclic form (PCF), which is found in the tse tse fly vector, and the bloodstream form (BSF), which is present in the blood of the infected animal host. Although both stages have a single mitochondrion, the PCF mitochondrion has a respiratory chain, while the BSF mitochondrion does not possess a functional respiratory chain or oxidative phosphorylation and relies on the reverse action of the ATP synthase to maintain a mitochondrial membrane potential (1–4) required for protein (5) and Ca^2+^ (2) transport. Both stages have a functional mitochondrial Ca^2+^ uniporter (MCU) (6–8), which is essential for growth and virulence (9).

Trypanosomes have significant differences in the composition and function of the MCU complex (MCUC), compared to mammalian cells: we have found paralogs of trypanosome MCU that we named MCUb, MCUc, and MCUd that are necessary for mitochondrial Ca^2+^ transport; MCUc and MCUd are present only in trypanosomatids (10, 11). These subunits form, together with MCU and MCUb, hetero-oligomeric complexes in membranes (10), in contrast to the *in vitro* homotetramers of recombinant MCU described for fungi (12–15) and zebra fish (15) and to the homopentamers described for Caenorhabditis elegans (16). The MCUb subunit is a Ca^2+^-conducting subunit and does not have a dominant negative effect on the channel like its mammalian ortholog (17). The mitochondrial calcium uptake 1 (MICU1) and MICU2 proteins do not have a gatekeeper function at low Ca^2+^ concentrations (18), as occurs with the mammalian orthologs (19–25). Finally, the trypanosomatid genomes lack orthologs encoding subunits present in the mammalian MCU complex, like MCU regulator 1 (MCUR1) (26) and essential MCU regulator (EMRE) (27).

The mitochondrial ATP synthase of T. brucei is a large multisubunit protein that is composed of two oligomeric components, a peripheral hydrophilic F_1_ complex and a base piece/stalk F_o_ complex, and contains additional subunits with no obvious homology to proteins outside the kinetoplastids (28). As in the mammalian enzyme, the F_1_ domain has three catalytic sites, while the hydrophobic F_o_ complex is embedded in the inner mitochondrial membrane and contains a proton channel (29). In mammalian mitochondria, the ATP synthase is attached to both the phosphate carrier (PiC) and the adenine nucleotide translocator (ANT), forming the so-called ATP synthasome (29, 30). A similar association of the ATP synthase with the adenine nucleotide carrier was also reported for Leishmania mexicana mitochondria (31) but not for T. brucei PCF mitochondria (32). The synthasome catalyzes the synthesis of ATP coupled to the mitochondrial entry of P_i_ by the phosphate carrier and the exchange of ADP for ATP by the adenine nucleotide translocator.

Calcium ion (Ca^2+^) is a key element in the pathway responsible for the activation of mitochondrial oxidative phosphorylation (33). In mammalian cells, intramitochondrial Ca^2+^ stimulates a pyruvate dehydrogenase phosphatase that activates the pyruvate dehydrogenase or allosterically activates 2-oxoglutarate and isocitrate dehydrogenases, resulting in increased ATP production (34–38). We found that trypanosomatid pyruvate dehydrogenase phosphatases are directly stimulated by Ca^2+^ (39). Ca^2+^ also increases the specific activity of the F_o_F_1_ ATP synthase in mammalian mitochondria (40), but as yet there is no clear mechanism to how Ca^2+^ activates the ATP synthase. The interaction of the MCU complex with the ATP synthasome would thus have physiological significance since this is a biological machine that couples ADP and P_i_ exchange with ATP production, a process that is stimulated by Ca^2+^. Here we report the direct physical interaction of the MCU complex with subunit c of the T. brucei, Trypanosoma cruzi, and human ATP synthases (TbATPc, TcATPc, and HsATPc, respectively), which is important for the bioenergetics of the cells.

## RESULTS

### Proteomic analysis of tandem-affinity-purified TbMCU complex.

**FIG 1:**
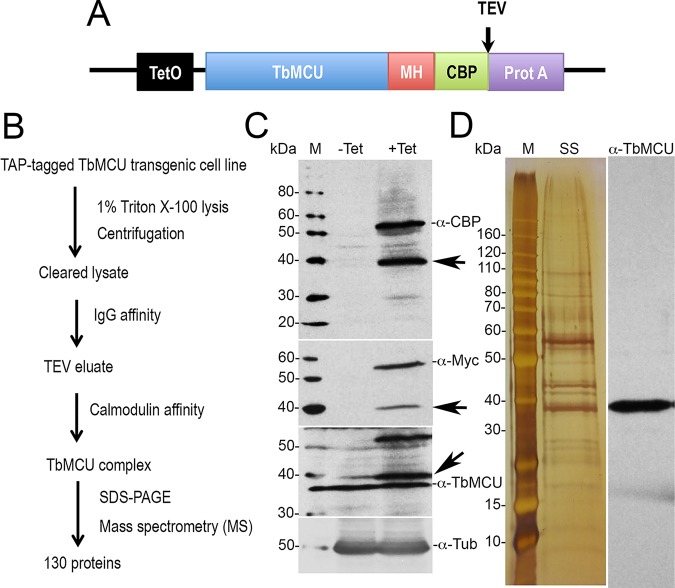
Tandem affinity purification (TAP) and proteomic analysis of TbMCU complex. (A) Diagram showing positions of tags. TEV, tobacco etch virus protease cleavage site; MH, Myc-His; CBP, calmodulin-binding peptide; Prot A, protein A domain. (B) Scheme of the TAP method used for isolation of TAP-tagged TbMCU complex. (C) Western blot analyses of tetracycline-inducible TAP-tagged TbMCU-TAP overexpressed in PCF trypanosomes against the CBP, MYC, and TbMCU antibodies. One additional band at approximately 40 kDa was detected, possibly because of degradation of the TAP-tagged TbMCU protein (arrows) by the AcTEV protease or an alternative translation termination at the TEV cleavage site. When anti-TbMCU antibody was used, both the TAP-tagged protein and the endogenous copy were detected. The blot was stripped and reincubated with antitubulin (α-Tub) antibody as a loading control. (D) SDS-PAGE of purified TbMCU complex (left, silver stained [SS]; right, immunoblotted with anti-TbMCU). M, PageRuler unstained protein ladder.

Tandem affinity purification (TAP) is a widely used method for the isolation of protein complexes under native conditions. We adapted the method developed by Panigrahi et al. (41) and modified by Jensen et al. (plasmid pLew79-MH-TAP) (42) using an inducible system to overexpress the T. brucei MCU (TbMCU) in the T. brucei PCF 29-13 cell line. The TbMCU subunit was fused to a TAP tag consisting of a c-MYC-His tag (MH) and a protein A domain separated from a calmodulin-binding peptide (CBP) by a tobacco etch virus (TEV) protease cleavage site (Fig. 1A). The sequential purification over IgG-Sepharose and calmodulin resin under native conditions (Fig. 1B to D) resulted in the mass spectrometry identification of 130 proteins (see Data Set S1A in the supplemental material), from two independent experiments. Of the proteins identified, the ones with the highest scores were TbMCU (Tb427tmp.47.0014) and 19 subunits (β, α, δ, OSCP, γ, p18, Tb1, Tb2, and 11 ATP synthase-associated proteins [28]) of the ATP synthase (Table 1). Interestingly, we also detected TbMCUb and TbMCUc, which we know interact with TbMCU (10), as well as voltage-dependent anion channel (VDAC), adenine nucleotide translocator (ANT), and phosphate carrier (PiC). In this regard, the F_o_F_1_ ATP synthase, ANT, and PiC form what is known as ATP synthasome (30). Similar mass spectral results were obtained by immunoprecipitation (IP) of T. brucei cells overexpressing TbMCU-hemagglutinin (HA) using anti-HA–agarose beads (Fig. S1 and Data Set S1B). Conspicuously absent were subunits a (encoded by the mitochondria) and c of the ATP synthase, although this could be expected giving their very hydrophobic nature and the need of special techniques for their chromatographic isolation and mass spectral detection (43).

**TABLE 1:** Proteins purified by TAP with TbMCU^*a*^

Accession no.	Mascot score	Length (aa)	Mass (kDa)	Description
Tb427.03.1380	2,838	519	55.7	ATP synthase subunit beta^*b*^
Tb427.07.7420	1,839	584	63.5	ATP synthase subunit alpha^*b*^
Tb427.06.3740	1,063	657	71.4	Heat shock 70-kDa protein^*b*^
Tb427.10.180	1,038	305	34.4	ATP synthase subunit gamma^*b*^
Tb427.10.8030	920	255	28.8	ATP synthase subunit OSCP^*b*^
Tb427.06.4990	820	182	20.1	ATP synthase subunit delta^*b*^
Tb427.05.1710	570	188	21.3	ATP synthase subunit p18
Tb427tmp.02.4120	534	269	27.6	ATP synthase-associated protein
Tb427.10.520	502	396	46.8	ATP synthase subunit Tb1^*b*^
Tb427tmp.47.0014	485	307	34.8	MCU^*b*^
Tb427.05.2930	472	370	43.3	ATP synthase subunit Tb2^*b*^
Tb427tmp.211.1750	372	317	34.3	PiC or TbMCP11
Tb427.10.300	296	254	28.4	MCUb^*b*^
Tb427tmp.01.4621	255	149	16.8	Calmodulin
Tb427tmp.03.0475	243	106	12.1	ATP synthase-associated protein
Tb427tmp.47.0022	226	169	20.2	ATP synthase-associated protein
Tb427.02.3610	207	144	16.1	ATP synthase-associated protein^*b*^
Tb427.10.14820	203	307	34.1	ANT or TbMCP5^*b*^
Tb427.02.2510	201	270	29.2	VDAC
Tb427tmp.02.1760	176	249	27.7	MCUc
Tb427.04.3450	161	114	13.7	ATP synthase-associated protein
Tb427.10.9830	147	157	17.2	ATP synthase-associated protein
Tb427.03.2880	121	104	12.6	ATP synthase-associated protein
Tb427.03.1690	78	145	17.1	ATP synthase-associated protein^*b*^
Tb427.03.2180	78	156	17.9	ATP synthase-associated protein^*b*^
Tb427.07.840	60	124	14.5	ATP synthase-associated protein
Tb427.05.3090	57	101	11.7	ATP synthase-associated protein

a Methods used and a complete list of proteins identified are available in the supplemental material (Text S1 and Data Set S1). aa, amino acids. Accession numbers are from TriTrypDB.

b Also identified by HA-tag IP (Fig. S1C).

10.1128/mBio.00268-20.2 FIG S1 Purification and mass spectrometry (MS) of HA-tagged TbMCU complex. (A) Scheme of the method used for isolation of HA-tagged TbMCU complex. (B) SDS-PAGE of purified TbMCU complex (left, silver stained; right, immunoblotted with anti-TbMCU). M, PageRuler unstained protein ladder. Arrows show two bands corresponding to TbMCU. The detection of two bands of tagged TbMCU has been shown before when tagging the protein (G. Huang, A. E. Vercesi, and R. Docampo, Nat Commun **4:**2865, 2013, https://doi.org/10.1038/ncomms3865) and could be due to interference of the tag with the processing of the N-terminal mitochondrial targeting signal. (C) Selected proteins purified by HA tag immunoprecipitation. Download FIG S1, PDF file, 0.8 MB. Copyright © 2020 Huang and Docampo. 2020 Huang and Docampo This content is distributed under the terms of the Creative Commons Attribution 4.0 International license.

10.1128/mBio.00268-20.9 DATA SET S1 (A) List of 130 proteins identified by TAP-LC MS/MS (Excel file). (B) List of 106 proteins identified by HA tag IP-liquid chromatography-tandem mass spectrometry (LC-MS/MS) (Excel file). Download Data Set S1, XLS file, 0.1 MB. Copyright © 2020 Huang and Docampo. 2020 Huang and Docampo This content is distributed under the terms of the Creative Commons Attribution 4.0 International license.

10.1128/mBio.00268-20.1 TEXT S1 Supplemental materials and methods. Download Text S1, DOCX file, 0.1 MB. Copyright © 2020 Huang and Docampo. 2020 Huang and Docampo This content is distributed under the terms of the Creative Commons Attribution 4.0 International license.

### Physical interaction of T. brucei ATP synthase subunit c with TbMCU.

As a “reverse” approach to identify the association of TbMCU with a potential ATP synthasome, we generated *in situ* HA-tagged T. brucei ATPβ (TbATPβ), TbATPp18 (a subunit that binds to each of the TbATPα subunits [44, 45]), TbANT, and TbPiC PCF cell lines. These proteins colocalized with MitoTracker (MT) to mitochondria of T. brucei PCF (Fig. S2A). Interestingly, TbMCU was immunoprecipitated by TbATPβ-HA or TbATPp18-HA, but not by TbANT-HA or TbPIC-HA, using anti-HA antibodies (Fig. S2B and C), suggesting that TbMCU is closely associated with the ATP synthase and probably is loosely associated with other components of the ATP synthasome.

10.1128/mBio.00268-20.3 FIG S2 Localization of HA-tagged TbATPβ, TbATPp18, TbANT or TbPiC to mitochondria of PCF and coimmunoprecipitation (co-IP) of TbMCU with those proteins using *in situ* HA-tagged PCF cell lines. (A) Colocalization of HA-tagged TbATPβ, TbATPp18, TbANT, or TbPiC with MT (PCCs of 0.8250, 0.8047, 0.6958, and 0.7917, respectively). DIC, differential interference contrast microscopy; MT, MitoTracker. Scale bars = 10 μm. The merged images indicate colocalization (in yellow). (B and C) Co-IP of TbMCU with TbATPβ, TbATPp18, TbANT, or TbPiC. Cell lysates from the HA-tagged TbATPβ, TbATPp18, TbANT, or TbPiC cell line were incubated with anti-HA antibody, and immunoprecipitates (IP) were resolved by SDS-PAGE. Input lysates (B) were blotted with antibodies against HA, while immunoprecipitates (C) were blotted with antibodies against TbMCU. M, molecular weight markers. The bait proteins (B) and the prey proteins (C) were detected by Western blot analysis with specific antibodies (indicated on the right), using TbCyt *c*_1_ as a control. Download FIG S2, PDF file, 1.2 MB. Copyright © 2020 Huang and Docampo. 2020 Huang and Docampo This content is distributed under the terms of the Creative Commons Attribution 4.0 International license.

**FIG 2:**
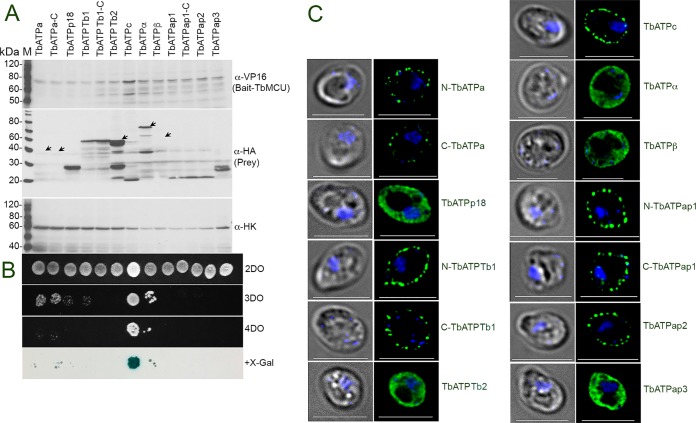
Physical interaction of TbATPc with TbMCU by split-ubiquitin membrane-based yeast two-hybrid (MYTH) assays. (A) Immunoblot validation of bait (TbMCU) and prey (ATP synthase subunits or associated proteins, as indicated) expression in yeast reporter strain NMY51. Lysates containing 60 μg of proteins from yeast transformants were subjected to SDS-PAGE on 10% polyacrylamide gels and transferred to a nitrocellulose membrane. The blots were probed with antibodies against VP16 (for bait), HA (for prey), and hexokinase (for a loading control). The weak or specific bands are indicated with arrows. Under the control of the same *CYC1* promoter, the preys demonstrated different expression levels, reflecting their genetic difference. (B) Growth of yeast reporter strain NMY51 expressing bait TbMCU, together with each prey (N-TbATPa, C-TbATPa, TbATPp18, N-TbATPTb1, C-TbATPTb1, TbATPTb2, TbATPc, TbATPα, TbATPβ, N-TbATPap1, C-TbATPap1, TbATPap2, or TbATPap3) on SD selection agar plates (SD-2DO, SD-3DO, SD-4DO, and SD-4DO plus X-Gal). Exponentially growing cells were harvested and resuspended at 5 × 10^6^ cells/ml, and 3 μl of a 1:10 dilution was spotted on an SD agar plate and incubated at 30°C for 2 to 3 days. A blue colony growing on a plate with high-stringency SD-4DO plus X-Gal confirmed the interaction between TbMCU and ATP synthase subunit c. (C) Fluorescence microscopy images validated proper yeast plasma membrane or cytosolic localization of the prey N-TbATPa-, C-TbATPa-, TbATPp18-, N-TbATPTb1-, C-TbATPTb1-, TbATPTb2-, TbATPc-, TbATPα-, TbATPβ-, N-TbATPap1-, C-TbATPap1, TbATPap2-, or TbATPap3-NubG-HA. Scale bars = 5 μm; left images are by differential interference contrast (DIC).

**FIG 3:**
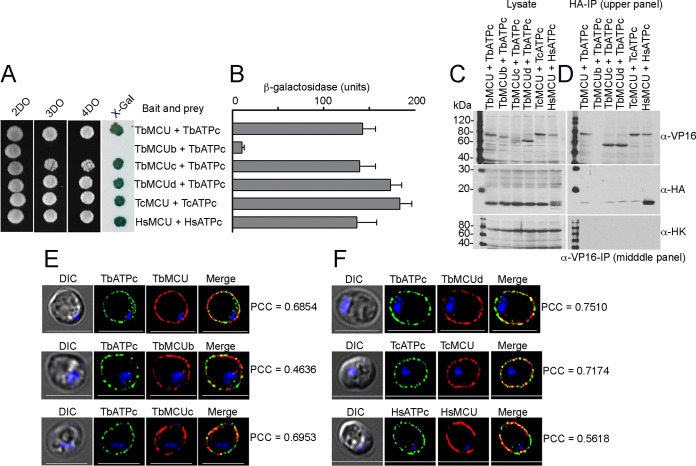
Direct physical MCU-ATPc interaction is conserved in trypanosomes and human cells. (A) Growth of yeast strain NMY51 expressing bait (TbMCU, TbMCUb, TbMCUc, TbMCU, TcMCU, or HsMCU) together with prey (TbATPc, TcATPc, or HsATPc) on SD selection agar plates (SD-2DO, SD-3DO, SD-4DO, and SD-4DO plus X-Gal) as described for Fig. 2B. (B) Quantitative β-galactosidase (β-Gal) activity assay determined the strength of interaction of MCU with ATPc. The yeast strain NMY51 coexpressing MCU bait and ATPc prey as described for panel A was used in a β-Gal microplate assay using *o*-nitrophenyl-β-d-galactopyranoside (ONPG) as the substrate. Each column represents mean ± standard deviation (*n* = 3; 6 colonies each independent experiment). (C and D) Coimmunoprecipitation (co-IP) validation of interactions between MCU and ATPc as indicated. Yeast lysates from the transformants were incubated with anti-VP16 or anti-HA antibodies, immunoprecipitates were resolved by SDS-PAGE, and input lysates (C) and immunoprecipitates (D) were blotted with antibodies against VP16, HA, and hexokinase (HK). Immunoblotting of the input lysates confirmed both bait and prey expression (as indicated). Hexokinase was used as a negative control. (E and F) Immunofluorescence validation of colocalization of the bait-prey interaction pairs as in panel A to the yeast plasma membrane. Pearson coefficient correlations (PCCs) of the bait-prey pairs TbMCU-TbATPc, TbMCUb-TbATPc, TbMCUc-TbATPc, TbMCUd-TbATPc, TcMU-TcATPc, and HsMCU-HsATPc are indicated. Left images are DIC. Scale bars = 5 μm. The merge images indicate colocalization in yellow.

In order to validate the interaction of TbMCU with the ATP synthase, we used split-ubiquitin membrane-based yeast two-hybrid (MYTH) assays (46) to determine the direct physical interactions between TbMCU and ATP synthase subunits in yeast. We followed the method that we used previously to determine the interaction among TbMCU, TbMCUb, TbMCUc, and TbMCUd (10). The split-ubiquitin system allows detection of *in vivo* interaction between membrane proteins that have their N and/or C terminus located in the cytosol. The membrane topology predicted by Protter of five ATP synthase membrane subunits or associated proteins showed that these membrane proteins could be localized to the yeast plasma membrane with either the N or C termini facing the cytosol (Fig. S3). In the MYTH assays, TbMCU (the bait, without the mitochondrial targeting signal [MTS]) is fused to the C-terminal half of ubiquitin (Cub) and the artificial transcription factor LexA-VP16 (TF), as described previously (10). Each of the 10 ATP synthase subunits selected (a [synthesized with yeast optimized codons], p18, Tb1, Tb2, c, α, β, and 3 associated proteins) (the prey, without MTS) was fused to the mutated half of ubiquitin (NubG), and the interaction of the protein partners was monitored by the release of the TF, which translocates to the nucleus, where it binds to LexA operators situated upstream of reporter genes (*HIS3*, *ADE2*, and *lacZ*) via its Lex DNA binding domain. The reporter genes enable the yeast to grow on defined media lacking histidine or/and adenine, while *lacZ* encodes the enzyme β-galactosidase (β-Gal), resulting in growth of yeast in selective medium and color development in β-Gal assays (Fig. 2B). TbMCU with the yeast invertase (SUC) signal sequence instead of MTS was expressed in pBT3-SUC as bait and targeted correctly to the yeast plasma membrane (10) (Fig. 3E). The 10 ATP synthase subunits were expressed in pPR3N or pPR3C as preys and targeted correctly to the yeast plasma membrane or cytosol as predicted (Fig. 2C). The yeast reporter strain expressing the bait TbMCU alone did not grow on the selective synthetic dropout (SD) plates (SD medium with a triple dropout [SD-3DO], SD-4DO, and SD-4DO plus X-Gal [5-bromo-4-chloro-3-indolyl-β-d-galactopyranoside]), indicating that the bait was not self-activated (10). Surprisingly, the strain expressing TbMCU as bait and only ATP synthase subunit c as prey enabled growth on the high-stringency selective SD-4DO plates and had high X-Gal activity (Fig. 2B), suggesting that TbMCU interacts strongly with ATP synthase subunit c. Expression of each of the bait-prey pairs in yeast was confirmed by Western blot analyses (Fig. 2A) using antitag antibodies from the MYTH expression vectors pBT3-SUC and pPR3N or pPR3C (43), anti-VP16 antibody for the bait, and anti-HA antibody for the prey, respectively.

10.1128/mBio.00268-20.4 FIG S3 Topology models of MCU and ATP synthase subunits or associated proteins (ap). Putative topology models of T. brucei ATP synthase subunits or associated proteins (TbATPa, TbATPTb1, TbATPc, TbATPap1, and TbATPap2), T. cruzi MCU (TcMCU), and Homo sapiens ATP synthase subunit c (HsATPc) and MCU (HsMCU), predicted with Protter. Putative mitochondrial targeting sequences (MTS) of these membrane proteins, predicted by MitoProt, are marked in red. TbATPa is encoded by the mitochondria of T. brucei. IMS, intermembrane space. Download FIG S3, PDF file, 0.6 MB. Copyright © 2020 Huang and Docampo. 2020 Huang and Docampo This content is distributed under the terms of the Creative Commons Attribution 4.0 International license.

### Direct physical MCU-ATPc interaction is conserved in trypanosomes and humans.

Like mammals, trypanosomes have 3 isoforms of ATP synthase subunit c. These isoforms differ in their cleavable MTS, whereas their mature proteins are identical in both T. brucei and T. cruzi (Fig. S4A). Since both MCU and ATP synthase subunit c are well conserved in most eukaryotes (47, 48), we investigated whether MCU-ATPc interaction was conserved in T. cruzi and human cells. We also investigated whether the other subunits of the TbMCU complex interacted with ATP synthase subunit c. TbMCUC subunits (TbMCU, TbMCUb, TbMCUc, and TbMCUd), T. cruzi MCU (TcMCU), and Homo sapiens MCU (HsMCU) were expressed as baits for MYTH assays, while TbATPc (identical to TcATPc) and HsATPc (Fig. S4B) were expressed as preys. Remarkably, each TbMCUC subunit, excluding TbMCUb, strongly interacted with TbATPc, TcMCU interacted with TcATPc, and HsMCU interacted with HsATPc (Fig. 3A and B). The MCU-ATPc interactions were confirmed by reciprocal coimmunoprecipitations (co-IP) (Fig. 3C and D) and immunofluorescence subcellular colocalization (Fig. 3E and F) using antitag antibodies, anti-VP16 for the bait, and anti-HA for the prey. The lack of TbMCUb-TbATPc interaction was consistent with the absence of coimmunoprecipitation in yeast lysates and also with lack of colocalization of these proteins in the yeast plasma membrane (Pearson coefficient correlation [PCC] of 0.4638), while there was colocalization of TbATPc with TbMCU, TbMUCc, and TbMCUd and of TcATPc and HsATPc with TcMCU and HsMCU, respectively (Fig. 3E and F). However, we cannot rule out that a different topology of TbMCUb could be responsible for this negative result.

10.1128/mBio.00268-20.5 FIG S4 Multiple-sequence alignment. (A) Sequences of deduced amino acids of TbATPc1 (TriTrypDB Tb427tmp.02.2950), TbATPc2 (Tb427.10.1570), and TbATPc3 (Tb427.07.1470) were aligned with MUSCLE (https://www.ebi.ac.uk/Tools/msa/muscle) and the webserver site (http://www.bioinformatics.org/sms/index.html). Identical (black) and similar (gray) amino acid residues are shaded. The groups of similar amino acids (ILV, FWY, KRH, DE, GAS, P, C, and TNQM) were used for the similarity calculation. Mitochondrial targeting sequences (MTS) are boxed in red. (B) Sequences of mature TbATPc and HsATPc (GenBank accession no. NP_001002027) proteins were aligned as described for panel A. The two putative transmembrane domains (TM1 and TM2), predicted by TMHMM Serve v. 2.0 (http://www.cbs.dtu.dk/services/TMHMM/), are overlined. The highly conserved glycine (GXGXGXG) motifs in TM1 required for c ring formation are boxed in red. The trimethylated lysine (K) in the loop and glutamate (E) in TM2, essential for proton translocation, are boxed in blue and green, respectively. Download FIG S4, PDF file, 1.3 MB. Copyright © 2020 Huang and Docampo. 2020 Huang and Docampo This content is distributed under the terms of the Creative Commons Attribution 4.0 International license.

### TMHs are determinants of the interactions between TbMCU and TbATPc.

**FIG 4:**
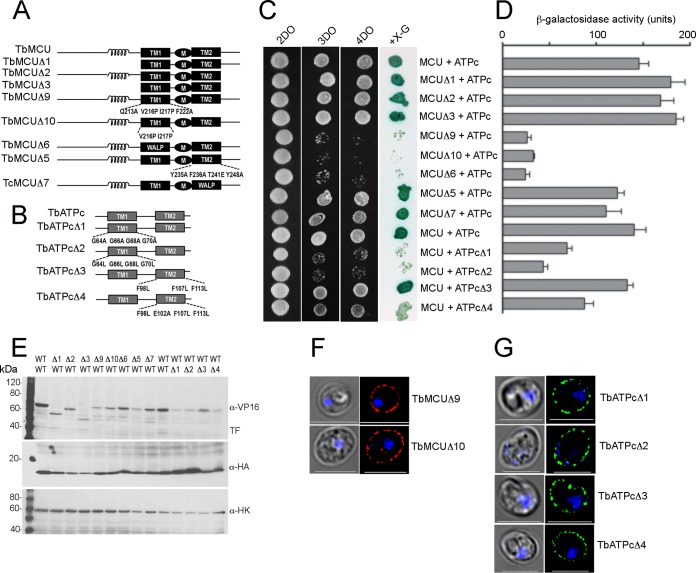
Determination of specific interactions between the transmembrane domains of TbMCU and TbATPc by mutagenesis and MYTH analyses. (A and B) The scheme depicts the wild-type (WT) and truncated and/or substitution mutant constructs of TbMCU (A) and TbATPc (B). Coil, coiled-coil domain; TM1 and TM2 (black or gray rectangles), transmembrane domains 1 and 2; M, the conserved WDXXEPXTY motif; WALP, artificial TM sequence GWWLALALALALALALWWA. Substitutions of the conserved residues of TMH1 or TMH2 of TbMCU or TbATPc are indicated (multiple substitutions were generated, because single substitutions did not significantly alter protein-protein interaction). (C) Growth assay of the yeast NMY51 strain expressing the bait (TbMCU WT and Δ1 to Δ3, Δ5, Δ7, Δ9, or Δ10 mutant) together with the prey (TbATPc WT or Δ1 to Δ4 mutant) on SD selection agar plates as described for Fig. 3A. (D) Quantitative β-Gal activity assay of strain NMY51 coexpressing the bait-prey pairs as described for panel C determined their interaction strength. Each column represents the mean ± standard deviation (*n *= 3; 6 colonies for each independent experiment). (E) Expression level of each bait or prey as determined by immunoblot analysis using antitag antibodies, VP16 for the bait and HA for the prey, and hexokinase antibodies used as a loading control. TF, transcription factor (*LexA-VP16*) cleavage (as indicated). First lane, molecular weight markers. (F and G) Fluorescence microscopy images validated proper yeast plasma membrane localization of expressed new baits TbMCUΔ9 and -Δ10 (F) and preys TbATPcΔ1 to -Δ4 (G). Left images are DIC. Scale bars = 5 μm.

To identify specific interacting domains or motifs of TbMCU that mediate interaction with TbATPc, TbMCU mutants with truncations or substitutions (Fig. 4A) were generated and expressed as baits for MYTH assays, as described previously (10). Deletion of the N- or/and C-terminal regions of TbMCU (TbMCUΔ1, TbMCUΔ2, and TbMCUΔ3) and mutations of the conserved residues in transmembrane helix 2 (TMH2) (Y235A, F236A, T241E, and Y248A) (TbMCUΔ5) or substitution of TMH2 with the artificial transmembrane “WALP” helix (TbMCUΔ7) did not affect interaction with the TbATPc (Fig. 4C and D), suggesting that the regions flanking the TMHs of TbMCU and the TMH2 are not involved in the protein-protein interaction. In contrast, TbMCU mutations of the conserved residues in TMH1 (Q213A, V216P, I217P, and F222A or V216P and I217P), named TbMCUΔ9 and TbMCUΔ10, or replacement of TMH1 with the artificial transmembrane WALP helix (TbMCUΔ6) significantly reduced the interaction with TbATPc (Fig. 4C and D), indicating that the conserved residues V216 and I217 of TMH1 of TbMCU are essential for TbMCU interaction with TbATPc.

Since TbATPc is embedded in the inner mitochondrial membrane and has short mitochondrial intermembrane N- and C-terminal regions, we tested the interactions between its TMHs with TbMCU. To define specific interacting residues or motifs of TbATPc that mediate this interaction, 4 substitution TbATPc mutants (Fig. 4B) were generated and expressed as preys for MYTH assays. TbATPc mutations of the conserved residues in TMH2 (F98L, F107L, and F113L, designated TbATPcΔ3, and mutations F98L, E102A, F107L, and F113L, designated TbATPcΔ4), did not affect or slightly affected interaction with the TbMCU (Fig. 4C and D), suggesting that TMH2 of TbATPc is not involved in the protein-protein interactions. In contrast, TbATPc mutations of the conserved residues in the TMH1 (G64A/L, G66A/L, G68/A/L, and G70A/L), named TbATPcΔ1 and TbATPcΔ2, significantly reduced interaction with TbMCU (Fig. 4C and D), indicating that the highly conserved glycine (GXGXGXG) motif in the TMH1 (Fig. S4B) is important for TbATPc interaction with TbMCU.

Expression of the bait-prey pairs in yeast lysates was confirmed by Western blot analyses using anti-VP16 and anti-HA antibodies to detect the baits and preys, respectively (Fig. 4E). The substitutions in the newly generated TbMCU bait mutants (TbMCUΔ9 and TbMCUΔ10) and the TbATPc prey mutants (TbATPcΔ1, TbATPcΔ2, TbATPcΔ3, and TbATPcΔ4) did not alter their plasma membrane localization in yeast, as detected by immunofluorescence microscopy (Fig. 4F and G). Collectively, the mutagenesis and MYTH assays suggested that TMH1s were determinants of the specific interactions between TbMCU and TbATPc.

### TMH1-TMH1 mediates specific interaction between trypanosomal MCU and ATPc.

**FIG 5:**
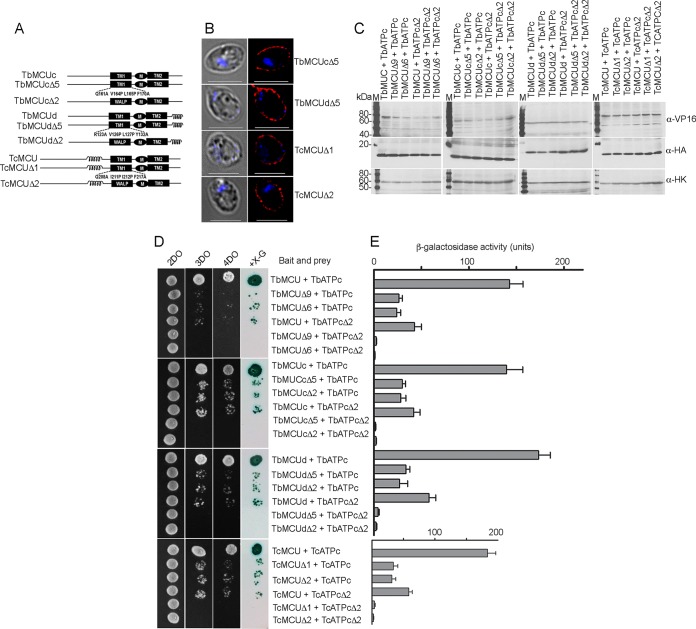
Verification of specific interactions between the TMH1s of TbMCU subunits or TcMCU and TbATPc by mutagenesis and MYTH analyses. (A) The scheme depicts the WT and substitution mutant constructs of TbMCUc, TbMCUd, and TcMCU as described for Fig. 4A. (B) Fluorescence microscopy images validated proper yeast plasma membrane localization of expressed new substitution bait TbMCUcΔ5, TbMCUdΔ5, or TcMCUΔ1 or -Δ2. Left images are DIC. Scale bars = 5 μm. (C) Expression level of each bait or prey as determined by immunoblot analysis using antitag antibodies, VP16 for the bait, HA for the prey, and hexokinase antibodies used as a loading control. Lanes M, molecular weight markers. (D) Growth assay of the yeast NMY51 strain expressing the bait (TbMCU WT or Δ6 or Δ9 mutant, TbMCUc WT or Δ2 or Δ5 mutant, TbMCUd WT or Δ2 or Δ5 mutant, and TcMCU WT or Δ1 or Δ2 mutant) together with the prey (TbATPc WT or Δ2 mutant) on SD selection agar plates as described for Fig. 3A. (E) A quantitative β-Gal activity assay of strain NMY51 coexpressing the bait-prey pairs as described for panel D determined their interaction strength. Each column represents the mean ± standard deviation (*n *= 3; 6 colonies for each independent experiment).

To investigate whether the TMH1s also mediate specific interactions between other T. brucei MCUC subunits (TbMCUc and TbMCUd) and TbATPc and between TcMCU and TcATPc, the TMH1 substitution mutants of TbMCUc (TbMCUcΔ5 and TbMCUcΔ2), TbMCUd (TbMCUdΔ5 and TbMCUdΔ2), and TcMCU (TcMCUΔ1 and TcMCUΔ2) were used (10) or newly generated and expressed as baits for MYTH assays, while the TMH1 substitution mutants of TbATPc (TbATPcΔ1 and TbATPcΔ2, identical to TcATPcΔ1 and TcATPcΔ2, respectively) were expressed as preys (Fig. 5A). The substitutions of newly generated MCU baits (TbMCUcΔ5, TbMCUdΔ5, TcMCUΔ1, and TcMCUΔ2) did not change their plasma membrane localization, as detected by immunofluorescence microscopy (Fig. 5B). Similar to the TbMCU-TbATPc interaction (Fig. 4C and D and Fig. 5D and E), the substitutions of TMH1 of TbMCUc, TbMCUd, or TcMCU or the substitution of TMH1 of TbATPc significantly disrupted their interactions, and the protein-protein interactions were completely blocked when both TMH1s of MCU and ATPc were mutated (Fig. 5D and E). These results suggest that TMH1 of each TbMCUC subunit or TcMCU specifically interacts with TMH1 of the TbATPc via the conserved residues or motifs of the TMH1s. Expression of the bait-prey pairs in yeast lysates was confirmed by Western blot analyses using anti-VP16 and anti-HA antibodies to detect the baits and preys, respectively (Fig. 5C).

### TbMCU complex interacts with the ATP synthase of T. brucei.

**FIG 6:**
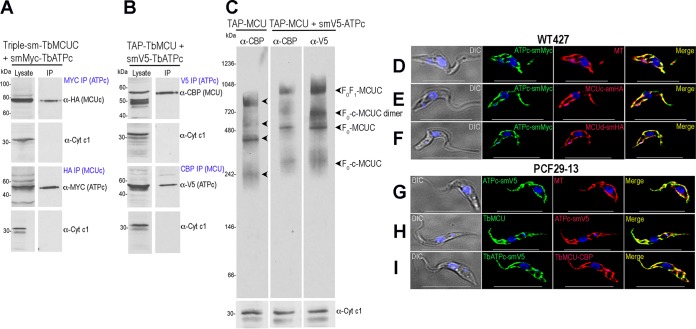
Coimmunoprecipitation and colocalization of TbMCU complex with TbATPc using *in situ* smFP-tagged ATPc T. brucei cell lines. (A and B) Coimmunoprecipitation of TbMCU complex (TbMCUC) with smMYC- or smV5-tagged TbATPc. (A) Cell lysates from the quadruple smFP-tagged (triple-smFP-tagged TbMCUC plus smMYC-tagged TbATPc) cell line were incubated with anti-MYC or anti-HA antibody, immunoprecipitates were resolved by SDS-PAGE, and input lysates and immunoprecipitates were blotted with antibodies against HA, MYC, or TbCyt *c*_1_. The bait proteins (left column, labeled as “Lysate” at the top) and the prey proteins (right column, labeled as “IP” at the top) were detected by Western blotting with the specific antibodies (indicated on the right) using TbCyt *c_1_* as a control. (B) Anti-V5, anti-CBP, and anti-TbCyt *c*_1_ immunoprecipitations were performed using lysates from the TAP-tagged TbMCU plus smV5-tagged TbATPc cell line, and input lysates and immunoprecipitates were blotted with antibodies against CBP, V5, and TbCyt *c*_1_. The bait and prey proteins were detected and shown as described for panel A. (C) BN-PAGE analyses of crude PCF mitochondrial vesicles from the TAP-tagged TbMCU and TAP-tagged TbMCU plus smV5-tagged TbATPc cell line. Immunoblot analyses were performed using antibodies against CBP or V5. Antibodies against TbCyt *c*_1_ were used as a loading control but detected on an SDS-PAGE gel. Arrowheads indicate four dominant bands at 250 to 900 kDa, representing F_o_F_1_ ATP synthase-MCUC (F_0_F_1_-MCUC), F_o_-c ring-TbMCUC (F_o_-c-MCUC) dimer, F_o_ ATP synthase-TbMCUC (F_o_-MCUC), and F_o_-c ring-TbMCUC (F_0_-c-MCUC), respectively. Markers are shown on the left, and the antibodies used in immunoblots are shown at the top. The similar patterns of protein complexes (arrowheads) were detected but shifted approximately 160 kDa in size (from the left blot to the middle and right blots) when TbATPc was tagged with smV5 (with a molecular weight of 44.2 kDa), indicating that 4 tagged subunits c were probably incorporated into the complexes of the smV5-tagged TbATPc cell line (middle and right blots). (D to F) Colocalization of smMYC-tagged TbATPc with MitoTracker (MT) (D), with smHA-tagged TbMCUc (E), and with smV5-tagged TbMCUd (F) (PCCs of 0.8500, 0.7425, and 0.8110, respectively). (G to I) Colocalization of smV5-tagged TbATPc with MT (G), with TbMCU (H), and with TAP-tagged TbMCU (I) (PCCs of 0.8771, 0.8339, and 0.8390, respectively). DIC, differential interference contrast microscopy. Scale bars, 10 μm. The merged images indicate colocalization (in yellow).

To confirm the TbMCU-TbATPc interaction in T. brucei, we overexpressed HA-tagged TbATPc1, TbATPc2, or TbATPc3 in PCF 29-13 cell lines (Fig. S5). The 3 isoforms of TbATPc colocalized with TbMCU to the mitochondria of PCF, as determined using anti-HA and anti-TbMCU antibodies (Fig. S5A). TbMCU was immunoprecipitated with the 3 TbATPc isoforms using anti-HA antibodies (Fig. S5B). To confirm that the TbMCU complex interacts with TbATPc, we also generated *in situ* smMYC-tagged or smV5-tagged TbATPc1 in the previously generated triple-smFP-tagged TbMCUC PCF cell line (10) and in the TAP-tagged TbMCU 29-13 cell line generated in this work, respectively. Coimmunoprecipitations (Fig. 6A and B) and colocalizations (Fig. 6D to I) of TbMCUC subunits (TbMCUc, TbMCUd, or TbMCU) with TbATPc revealed that TbMCU complex interacts with TbATPc in T. brucei. Blue native PAGE (BN-PAGE) and immunodetection analyses with anti-CBP and anti-V5 antibodies indicated that the TbMCU complex physically interacts with mitochondrial ATP synthase subunit c in a large protein complex with a molecular weight of approximately 900 kDa (Fig. 6C). This large protein complex possibly associates with TbANT and TbPiC to form a potential “ATP synthasome” in T. brucei.

10.1128/mBio.00268-20.6 FIG S5 Colocalization and coimmunoprecipitation of TbMCU with TbATPc using HA-tagged TbATPc1-, TbATPc2-, or TbATPc3-overexpressing cell lines. (A) Colocalization of HA-tagged TbATPc1, TbATPc2, or TbATPc3 with TbMCU (PCCs of 0.8896, 0.9251, and 0.8599, respectively). Scale bars = 10 μm. The merged images indicate colocalization (in yellow). (B) Co-IP of TbMCU with TbATPc1, TbATPc2, or TbATPc3. Cell lysates from the HA-tagged TbATPc1-, TbATPc2-, or TbATPc3-overexpressing cell line were incubated with anti-HA antibody, and immunoprecipitates were resolved by SDS-PAGE. Input lysates (left) were blotted with antibodies against HA, while immunoprecipitates (right) were blotted with antibodies against TbMCU. The bait proteins (left) and the prey proteins (right) were detected, respectively, by Western blot analysis with specific antibodies (indicated on the right) using TbCyt *c*_1_ as controls. The upper bands with molecular weights of approximately 45 kDa and 100 kDa (left) are TbATPc oligomers as indicated. Protein bands corresponding to immunoglobulin heavy (H) and light (L) chains (right) are indicated. M, molecular weight marker. Download FIG S5, PDF file, 1.3 MB. Copyright © 2020 Huang and Docampo. 2020 Huang and Docampo This content is distributed under the terms of the Creative Commons Attribution 4.0 International license.

To obtain additional evidence supporting the interaction of the TbMCU complex with the TbATP synthase of T. brucei, we generated *in situ* smMYC-tagged or smV5-tagged TbATPβ and TbATPp18 T. brucei PCF cell lines using the triple-smFP-tagged TbMCUC PCF wild-type (WT) cell line (10) and the TAP-tagged TbMCU PCF29-13 cell line, respectively. The smMYC-tagged or smV5-tagged TbATPβ/TbATPp18 colocalized with MitoTracker (MT) to mitochondria of PCF (Fig. S6A to D) and coimmunoprecipitated with TbMCUc and TbMCU (Fig. S6E and F), further confirming that the TbMCU complex interacts with the mitochondrial ATP synthase in T. brucei.

10.1128/mBio.00268-20.7 FIG S6 Localization and co-IP of smFP-tagged TbATPβ and TbATPp18 with TbMCU complex using *in situ*-tagged TbATPβ and TbATPp18 PCF cell lines. (A and B) Colocalization of smMYC-tagged TbATPβ (A) or TbATPb (B) with MT (PCCs of 0.7704 and 0.8467, respectively). (C and D) Colocalization of smV5-tagged TbATPβ (C) or TbATPp18 (D) with MT (PCCs of 0.9044 and 0.8977, respectively). Scale bars, 10 μm. The merged images indicate colocalization (in yellow). (E) Co-IP of TbMCU complex with TbATPβ or TbATPp18. Cell lysates from the quadruple-smFP-tagged (triple-smFP-tagged TbMCUC plus smMYC-tagged TbATPβ or TbATPp18) cell line were incubated with anti-MYC or anti-HA antibody, immunoprecipitates were resolved by SDS-PAGE, and input lysates and immunoprecipitates were blotted with antibodies against HA, MYC, or TbCyt *c*_1_. (F) Anti-V5, anti-CBP, and anti-TbCytc1 immunoprecipitations were performed using lysates from the TAP-tagged TbMCU plus smV5-tagged TbATPβ or TbATPp18 cell line, and input lysates and immunoprecipitates were blotted with the antibodies against CBP, V5, and TbCyt *c*_1_. The bait proteins (left 2 lanes of panels E and F, labeled as “Lysate” at the top) and the prey proteins (right 2 lanes of panels E and F, labeled as “IP” at the top) were detected by Western blot analysis with specific antibodies (indicated on the right) using TbCyt *c_1_* as a control. (G) Model showing submitochondrial localization and organization of putative MCUC-ATP synthasome “megacomplex” in trypanosomes. The MCU complex physically interacts with the ATP synthasome (ATP synthase, ANT, and PiC) via the c ring of F_o_ ATP synthase. In trypanosomes, ATP synthase consists of F_1_ moiety with the central stalk (α_3_β_3_, γ, δ, and ε) for ATP synthesis, F_0_ moiety with the putative stator (c_n_, a, p18, Tb1, Tb2, and OSCP) for proton (H^+^) translocation, and trypanosome-specific associated proteins (ap), while the molecular identity of the peripheral stalk is unknown. OSCP, oligomycin sensitivity-conferring protein; ANT, adenine nucleotide translocase; PiC, phosphate carrier. (H) Cross-section model of hypothetical T. brucei C_n_-ring-MCUC. The T. brucei heterohexameric MCUC consisting of 4 different subunits (MCU, MCUb, MCUc, and MCUd), with a molecular weight of approximately 145 kDa, is within the c ring of ATP synthase. TM1 of each MCUC subunit (excluding MCUb) interacts with TM1 of ATPc. The c ring rotates in a counterclockwise direction and translocates H^+^ from the intermembrane space to matrix during ATP synthesis. Download FIG S6, PDF file, 1.1 MB. Copyright © 2020 Huang and Docampo. 2020 Huang and Docampo This content is distributed under the terms of the Creative Commons Attribution 4.0 International license.

### Biological significance of the MCU-ATPc interaction in T. brucei.

**FIG 7:**
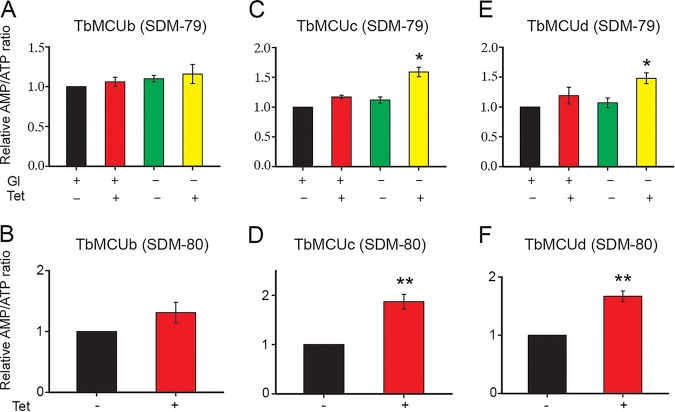
Changes in cellular AMP/ATP ratios after RNAi of *TbMCUb*, *TbMCUc*, and *TbMCUd*. (A, C, and E) Comparison of AMP/ATP ratios between PCF trypanosomes grown in the absence (−) or presence (+) of tetracycline (Tet) to induce RNAi of *TbMCUb* (A), *TbMCUc* (C), and *TbMCUd* (E), with or without glucose (Gl), expressed as fold increase (means ± standard deviations; *n *= 3; *, *P < *0.05, Student’s *t* test). (B, D, and F) Comparison of AMP/ATP ratios between PCF trypanosomes grown with and without tetracycline in a glucose-depleted medium (SDM-80) containing 5.2 mM l-proline for 2 days after 2 days of growth in SDM-79 with and without tetracycline, expressed as fold increase (means ± standard deviations; *n *= 3; **, *P < *0.01, Student’s *t* test).

The MCU-ATPc interaction provides evidence that the MCU complex forms part of a functional megacomplex including the ATP synthase, the ANT, and the PiC, which couples Ca^2+^ transport with ATP synthesis. We reported previously that downregulation of *TbMCU* increased the AMP/ATP ratio in T. brucei PCF grown in glucose-rich medium and that the ratio increased more significantly in the absence of glucose and presence of proline, when mitochondrial metabolism is more active (10). Similarly, ablation of other *TbMCUC* subunits (*TbMCUc* and *TbMCUd*), with exception of *TbMCUb*, by RNA interference (RNAi) resulted in an increased cellular AMP/ATP ratio in the absence of glucose and presence of proline, revealing the downregulation of ATP production in the cells (Fig. 7). These results are compatible with the coupling of Ca^2+^ transport with ATP synthesis.

### Specific interaction between HsMCU and HsATPc *in vitro* and *in vivo*.

**FIG 8:**
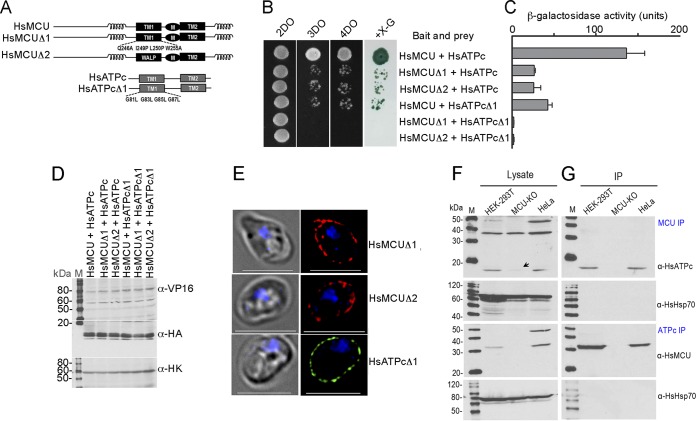
Specific interaction between HsMCU and HsATPc *in vitro* as determined by mutagenesis and MYTH assays and *in vivo* as assayed by coimmunoprecipitation. (A) The scheme depicts the WT and substitution mutant constructs of HsMCU and HsATPc as for Fig. 4A. (B) Growth assay of the yeast strain NMY51 expressing the bait (HsMCU WT or Δ1 or Δ2 mutant) together with the prey (HsATPc WT or Δ1 mutant) on SD selection agar plates as described for Fig. 3A. (C) A quantitative β-Gal activity assay of the strain NMY51 coexpressing the bait-prey pairs as described for panel B determined their interaction strength. Each column represents the mean ± standard deviation (*n *= 3; 6 colonies for each independent experiment). (D) Expression level of each bait or prey as determined by immunoblot analysis using antitag antibodies, VP16 for the bait, HA for the prey, and hexokinase antibodies used as a loading control. Lanes M, molecular weight markers. (E) Fluorescence microscopy images validated proper yeast plasma membrane localization of expressing bait HsMCUΔ1 or -Δ2 or prey HsATPcΔ1. Left images are DIC. Scale bars = 5 μm. (F and G) Coimmunoprecipitation of MCU and ATPc in human cells. Lysates from WT HEK-293T, MCU-KO, or HeLa cells were incubated with anti-HsMCU or anti-HsATPc antibodies, immunoprecipitates were resolved by SDS-PAGE, and input lysates (F) and immunoprecipitates (G) were blotted with antibodies against HsMCU, HsATPc, and HsHsp70. HsATPc was downregulated in the MCU-KO cells. Heat shock protein 70 (Hsp70) was used as a negative control. The bait proteins (F) and the prey proteins (G) were detected by Western blotting with the specific antibodies (indicated on the right of panel G) using human Hsp70 (HsHsp70) as a control. HsATPc was downregulated in the MCU-KO cells, as indicated by the arrow (F).

To determine whether TMH1s mediate the specific interaction between HsMCU and HsATPc, HsMCU and HsATPc mutants (designated HsMCUΔ1/Δ2 and HsATPcΔ1) were generated (Fig. 8A) and expressed as baits and preys, respectively, for MYTH assays. Similar to the interactions between TbMCU and TbATPc (Fig. 5D and E), replacements of TMH1s of HsMCU and HsATPc significantly disrupted or completely blocked their interactions (Fig. 8B and C), suggesting that the TMH1s of both HsMCU and HsATPc are essential for the protein-protein interaction while they did not alter their expression (Fig. 8D) or plasma membrane localization in yeast (Fig. 8E). To confirm the HsMCU-HsATPc interaction in human cells, we carried out reciprocal coimmunoprecipitations of HsMCU and HsATPc using HEK-293T and HeLa cells, with HEK-293T (MCU-KO) as a negative control. The cells were lysed (Fig. 8F) and immunoprecipitated under native conditions with anti-HsMCU or anti-HsATPc antibodies. HsATPc was pulled down with HsMCU using anti-HsMCU, and HsMCU was pulled down with HsATPc using anti-HsATPc, but human heat shock protein 70 (HsHsp70) was not immunoprecipitated with any of the antibodies (Fig. 8G). Furthermore, neither HsMCU nor HsATPc was pulled down from the MCU-KO lysate using these antibodies (Fig. 8G). These results confirm that HsMCU specifically interacts with HsATPc both in the yeast MYTH reporter strain and in human cells.

## DISCUSSION

Our studies revealed that three of the T. brucei MCU subunits (TbMCU, TbMCUc, and TbMCUd) physically interact with mitochondrial ATP synthase subunit c (TbATPc) when expressed in yeast membranes. This interaction, which is also observed with the T. cruzi subunits, is mediated by the conserved residues of TMH1s of both MCU and ATPc subunits and was confirmed by their coimmunoprecipitation in yeast lysates and by their colocalization, as detected by immunofluorescence analysis (IFA). The interaction of the TbMCUC with TbATPc was confirmed in trypanosomes *in vivo* by their coimmunoprecipitation from lysates of the parasite after the *in situ* tagging of the subunits, by their colocalization, as detected by IFA, and by blue native PAGE and immunodetection analysis. This interaction leads to the pulldown of the ATP synthase complex together with the adenine nucleotide translocator (ANT) and phosphate carrier (PiC) by TbMCU. This pulldown reveals the formation of a potential TbMCUC-ATP synthasome “megacomplex” important for the bioenergetics of the cells, as suggested by the increase in the AMP/ATP ratio after downregulation of the TbMCU subunits by RNAi (Fig. 7). We also demonstrated that this interaction is conserved in human cells when HsMCU and HsATPc are expressed in yeast membranes or when they are coimmunoprecipitated from HEK-293 and HeLa cell lysates using specific antibodies.

ATP synthase subunit c is very conserved and it arranges in the so-called c ring, which is an essential component of the F_o_ rotor (reviewed in reference 49). Although the number of c subunits forming the c ring is constant in a given species, it can be in the range 8 to 15, being smaller in eukaryotes than in prokaryotes (49). In eukaryotes, the inner helix (TMH1) has the highly conserved motif of four glycine residues (GXGXGXG) that we found is necessary for interaction with TMH1 of the MCU subunits of trypanosomes and human cells when expressed in yeast membranes. The outer helix (TMH2) has been proposed to transport H^+^ when the c ring rotates counterclockwise (viewed from the matrix) (50). Our MYTH results revealed that TMH1 of each TbMCU, except for TbMCUb, interacts with TMH1 of TbATPc, suggesting that, if this interaction occurs in mitochondria *in situ*, the TbMCU complex is within the c-ring of the ATP synthase in T. brucei (see Fig. S6G and H in the supplemental material). The c ring is reported to surround an internal phospholipid-containing cavity in bacteria (51), while a recent report on the cryo-electron microscopy (cryo-EM) structure of the porcine ATP synthase (52) identified a helical density in the center of the c_8_ ring that the authors attributed to subunit 6.8PL. However, they indicated that a structure with better-defined density will be needed to establish the identity of this protein (52). It would be interesting to know whether this helical density corresponds to the MCU.

It has not escaped our notice that the interaction between MCU and the c subunit immediately suggests a potential role for the MCU complex in the formation of the mitochondrial permeability transition pore (mPTP). The mPTP has long been considered a mediator of cell death mechanisms in mammals and as an alternative mechanism to the mitochondrial (intrinsic) pathway of apoptosis (53). The mPTP is a high-conductance nonselective channel located at contact sites between the inner and outer mitochondrial membranes. Its molecular composition is not yet clear, although several proteins have been shown to be components of this channel, including voltage-dependent anion channels (VDAC), ANT, PiC, cyclophilin D (CypD), and other proteins, such as spastic paraplegia 7 (SPG7) (54) and dimers of the ATP synthase (55). PTP opening can be enhanced by Ca^2+^ overload, oxidative stress, thiol oxidation, pyridine nucleotide oxidation, alkalinization, or low transmembrane potential and is inhibited by cyclosporine, which binds to cyclophilin D (55). Opening of this pore leads to mitochondrial dysfunction and cell death by either apoptosis or necrosis (53). Recent work on the components of the mPTP has implicated the c ring of the ATP synthase (56, 57), a channel inside ATP synthase dimers (55, 58), or the purified ATP synthase itself (59), as forming the pore. Interestingly, vestigial ATP synthases devoid of c subunits maintain mPTP formation features, suggesting that the c ring *per se* is not the channel (60).

Our results on the interaction of TbMCU with the ATP synthasome suggest that this “megacomplex” couples ADP and P_i_ transport with ATP synthesis, a process that is stimulated by Ca^2+^. Several subunits of ATP synthase appear to be Ca^2+^ regulated. Territo et al. (61, 62) suggested a direct activation of the ATPase by Ca^2+^ with a *K_m_* of 200 nM, well within the physiological range. Subunit c of F_o_ ATP synthase from chloroplasts and bacteria was identified as a calcium-binding protein and proposed to be involved in Ca^2+^ gating of the F_o_ proton (H^+^) channel (63). The catalytic β subunit of F_1_ complex from mammalian mitochondrial ATP synthase was also identified as a calcium-binding protein in two studies (64, 65). One proposed the Ca^2+^ binding as a potential mechanism for the Ca^2+^-dependent regulation of ATP synthesis (64), and the other suggested the binding as a trigger for the mitochondrial permeability transition (65).

Transient knockdown of *HsMCU* by small interfering RNA (siRNA) resulted in a 3-fold increase of the AMP/ATP ratio in HeLa cells (26). Our work shows that RNAi of *TbMCUc* or *TbMCUd* significantly increased the cellular AMP/ATP ratio, similar to what occurs upon knockdown/knockout of other trypanosome MCUC subunits in both T. brucei (66) and T. cruzi (11, 67). These results indicate that ATP production is tightly regulated by mitochondrial Ca^2+^ uptake in both trypanosomes and human cells.

In conclusion, coupling of MCU complex with the mitochondrial ATP synthasome is a novel mechanism for Ca^2+^-dependent regulation of ATP synthesis. Elucidation of the MCUC-ATP synthasome “megacomplex” will significantly advance our understanding of mitochondrial physiology.

## MATERIALS AND METHODS

### TAP-tagged TbMCU cell line.

To construct C-terminally TAP-tagged TbMCU for tandem affinity purification of the TbMCU complex, the full-length cDNA of *TbMCU* without the stop codon was amplified from T. brucei genomic DNA by PCR using the primers TbMCU-TAP-F and TbMCU-TAP-R (see Data Set S2A in the supplemental material), digested with BamHI and HindIII, and then cloned in frame into the enzyme-cut pLew79-MH-TAP vector (68) to generate pLew79-MH-TAP (*TbMCU*) (Data Set S2B). The recombinant construct pLew79-MH-TAP (*TbMCU*) was confirmed by sequencing at the DNA Analysis Facility at Yale University (New Haven, CT), NotI linearized, and then purified with Qiagen’s DNA purification kit for cell transfections. After transfection, phleomycin-resistant clones were selected and checked for tetracycline-regulated expression of TAP-tagged TbMCU (Fig. 1B), which is composed of a protein A domain separated from a calmodulin-binding peptide (CBP) sequence by a TEV protease cleavage site (Fig. 1A).

10.1128/mBio.00268-20.10 DATA SET S2 (A) Primers used in this study. (B) Plasmids constructed in this study. (C) Plasmids used in this study. (D) Antibodies used in this study. Download Data Set S2, XLSX file, 0.03 MB. Copyright © 2020 Huang and Docampo. 2020 Huang and Docampo This content is distributed under the terms of the Creative Commons Attribution 4.0 International license.

### Purification of epitope-tagged TbMCU complex.

TAP- or HA-tagged TbMCU was expressed by induction with tetracycline (200 ng/ml of culture) for 48 h. The tagged proteins and associated complexes were purified from 600 ml of cells harvested at a density of 2 × 10^7^ cells per ml by IP or tandem affinity chromatography (28, 41). First, the harvested cells (∼1.2 × 10^10^ cells in total) were washed once in phosphate-buffered saline (PBS) with 6 mM glucose and lysed with 1% Triton X-100 in 18 ml of ice-cold IPP150 (10 mM Tris-HCl [pH 8.0], 150 mM NaCl, 0.1% NP-40) with two dissolved tablets of Complete, EDTA-free protease inhibitor cocktail (Roche) on ice for 30 min. Next, the lysate was cleared of debris by centrifugation at 15,000 × *g* for 15 min at 4°C and the supernatant (cleared lysate) containing soluble proteins and tagged-TbMCU complex was further purified by using two complementary methods as described below.

TAP-tagged TbMCU complex was purified by a tandem affinity purification approach as previously described (28, 41), with some modifications (Fig. 1B). The cleared lysate from TAP-tagged TbMCU cell culture was incubated with 300 μl of IgG-Sepharose 6 Fast Flow beads (Pharmacia) overnight with gentle rotation at 4°C. The TAP-tagged proteins bound to IgG-Sepharose were washed three times in a Poly-Prep chromatography column (Bio-Rad) with 20 ml of IPP150, following equilibration by washing once in 10 ml of TEV cleavage buffer (IPP150, 0.5 mM EDTA, 1 mM dithiothreitol [DTT]). The protein-bead mix was resuspended in 1 ml of TEV buffer containing 10 μl of AcTEV protease (Invitrogen; 10 units/μl) and then incubated at 16°C for 2 h with constant mixing. After collection of the TEV eluate, beads were briefly washed with 0.5 ml of TEV buffer, and the washing was combined with the eluate. The total eluate (1.5 ml) containing tagged proteins and associated complexes was then diluted in 3 volumes of calmodulin binding buffer (IPP150, 10 mM fresh 2-mercaptoethanol, 1 mM magnesium acetate, 1 mM imidazole, 2 mM CaCl_2_) and 0.003 volume of 1 M CaCl_2_ and incubated with 200 μl of calmodulin resin (Stratagene). The mixture was incubated for 1 h with gentle rotation at 4°C and then washed in the same binding buffer. Fractions were eluted with EGTA elution buffer (same as calmodulin binding buffer but containing 2 mM EGTA instead of CaCl_2_). Eluted fractions were stored in aliquots at –80°C until use.

HA-tagged TbMCU complex was purified by an HA tag IP approach (Fig. S1A) using a Pierce HA tag IP/co-IP kit (Thermo Scientific) according to the manufacturer’s instructions. Briefly, the cleared lysate (supernatant) from an HA-tagged TbMCU cell line (9) was incubated with 200 μl of anti-HA–agarose slurry (∼350 μg of antibody) overnight with gentle rotation at 4°C. The agarose-bound complex was precipitated by centrifugation at 1,600 × *g* for 5 min at 4°C, resuspended in 850 μl of ice-cold IPP150 with 1× Complete, EDTA-free protease inhibitor cocktail (Roche), and then transferred to a Pierce spin column. The protein-agarose mix was centrifuged at 16,000 × *g* for 10 s, washed once in IPP150, and then washed three times in 0.5 ml of TBS-T (25 mM Tris-HCl, 0.15 M NaCl [pH 7.2], 0.05% Tween 20) by mixing and centrifugation. HA-tagged proteins and associated complexes were eluted in 50 μl of elution buffer (pH 2.8) by centrifugation and immediately neutralized by adding 2.5 μl of Tris (pH 9.5).

Full-size blots are shown in Fig. S7.

10.1128/mBio.00268-20.8 FIG S7 Full-size blots. Download FIG S7, PDF file, 0.4 MB. Copyright © 2020 Huang and Docampo. 2020 Huang and Docampo This content is distributed under the terms of the Creative Commons Attribution 4.0 International license.
