# Supplementary material for: The Mitochondrial Calcium Uniporter Interacts with Subunit c of the ATP Synthase of Trypanosomes and Humans
Source: mBio. 2020 Mar 17;11(2):e00268-20. doi: 10.1128/mBio.00268-20 (PMC7078472; doi:10.1128/mBio.00268-20)
Supplement: FIG S1 [file mBio.00268-20-sf001.pdf]

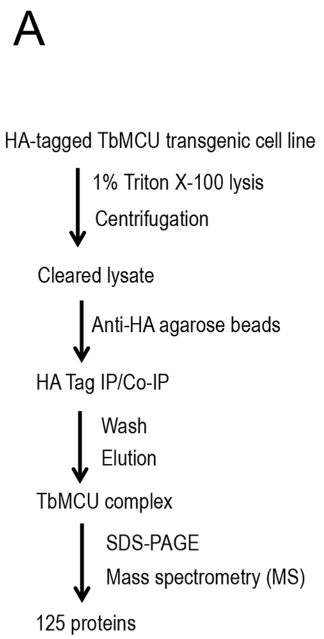

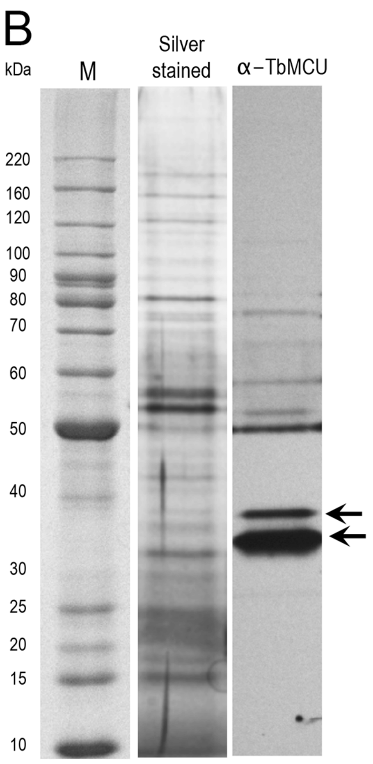

C

Proteins purified by HA Tag IP

Accession no.	Score	Description
Tb427tmp.47.0014	1469	MCU
Tb427.06.3740	903	Heat shock 70 kDa protein
Tb427.03.1380	693	ATP synthase subunit beta (β)
Tb427.07.7420	145	ATP synthase subunit alpha (α)
Tb427.10.14820		138 ANT or TbMCP5
Tb427.02.3610	128	ATP synthase associated protein
Tb427.06.4990	117	ATP synthase subunit delta (δ)
Tb427.10.180	106	ATP synthase subunit gamma (γ)
Tb427.10.8030	91	ATP synthase subunit OSCP
Tb427.10.520	74	ATP synthase subunit Tb1
Tb427.05.2930	43	ATP synthase subunit Tb2
Tb427.10.300	36	MCUb
Tb427.03.1690	35	ATP synthase associated protein
Tb427.03.2180	30	ATP synthase associated protein
