# Supplementary material for: The Mitochondrial Calcium Uniporter Interacts with Subunit c of the ATP Synthase of Trypanosomes and Humans
Source: mBio. 2020 Mar 17;11(2):e00268-20. doi: 10.1128/mBio.00268-20 (PMC7078472; doi:10.1128/mBio.00268-20)
Supplement: TEXT S1 [file mBio.00268-20-s0001.docx]

**SUPPLEMENTAL MATERIALS AND METHODS**

**Cell culture.** *T. brucei* PCF trypanosomes (427 and 29-13 strains) were used. PCF 29-13 trypanosomes (*T7RNAP NEO TETR HYG*) co-expressing T7 RNA polymerase and *Tet* repressor were a gift from Dr. George A. M. Cross (Rockefeller University, NY) (1) and were grown in SDM-79 medium (2), supplemented with hemin (7.5 µg/mL) and 10% heat-inactivated fetal bovine serum (FBS-HI), and at 27^o^C in the presence of G418 (15 µg/ml) and hygromycin (50 µg/ml) to maintain the integrated genes for T7 RNA polymerase and tetracycline repressor, respectively. HA-tagged TbMCU overexpressing PCF 29-13 cell line, *TbMCUb*, *TbMCUc* and *TbMCUd* RNAi PCF 29-13 cell lines and triple sm-FP-tagged TbMCUC PCF 427 cell line, which we generated previously (3, 4), were maintained in SDM-79 medium as described above with appropriate antibiotics (15 µg/ml G418, 50 µg/ml hygromycin, 10 μg/ml blasticidin or 5 μg/ml phleomycin). Wild-type HEK-293T cells, the MCU-KO cells (a gift from Drs. K. Kamer and V. K. Mootha, Harvard Medical School), and HeLa cells were grown in Dulbecco’s Modified Eagle Medium (DMEM) with 10% FBS-HI at 37^o^C.

**Chemicals and reagents.** MagicMark XP protein standard, PageRuler^TM^ unstained protein ladder, Mito-Tracker Red CMXRos, AcTEV protease, Alexa-conjugated secondary antibodies, mouse monoclonal antibody against V5, polyclonal rabbit antibody against HsATPc, NativePAGE Novex BIS-Tris Gel System (4-16%), Bis-Tris Gels, Sample Buffer, Running Buffer, 5% G-250 Sample Additive, Transfer Buffer, NativeMark Unstained Protein Standard, ultra X-β-Gal, Yeast β-galactosidase Assay Kit, Pierce BCA Protein Assay Kit, Pierce Silver Stain Kit, and Phusion High-Fidelity DNA polymerases, Phusion Site-Directed Mutagenesis Kit, Enhanced chemiluminescence (ECL) detection kit, Pierce Protein A/G Plus Agarose, Pierce HA Tag IP/Co-IP kit, and Pierce ECL Western blotting substrate were purchased from Thermo Fisher Scientific Inc. (Rockford, IL). Difco yeast nitrogen base w/o amino acids, and In-Fusion HD Cloning kit were purchased from Clontech (Mountain View, CA). Restriction endonucleases were purchased from New England Biolabs (Ipswich, MA). Complete, EDTA-free protease inhibitor cocktail tablets were purchased from Roche Applied Science (Indianapolis, IN). X-gal was purchased from Promega (Madison, WI). The pLEW100HA-BSD vector (Data Set S2C) was described before (4). The pMOTag4H vector was a gift from Dr. Thomas Seebeck (University of Bern, Bern, Switzerland) (5). The pLew79-MH-TAP vector was a gift from Dr. Marilyn Parsons (Seattle Children’s Research Institute, Seattle, WA) (6). The plasmid pMOTag5H was obtained from Addgene (Cambridge, MA). The pCAG_smFP c-MYC plasmid was a gift from Dr. Loren I. Looger (HHMI, Ashburn, VA) (7). The pBT3-SUC and pPR3N plasmids and yeast NMY51 MYTH reporter strain (8) were obtained from Creative Biolabs (NY, USA). The Bradford protein assay reagent, Poly-Prep Chromatography Column, and nitrocellulose membranes were from Bio-Rad (Hercules, CA). Immobilon-P PVDF membrane was from Millipore (Billerica, MA). QIAquick gel extraction kit and MinElute PCR purification kit were from Qiagen (Valencia, CA). CSM-Leu, CSM-Trp, CSM-Trp-Leu, CSM-Leu-Trp-His, CSM-Leu-Trp-His-Ade, yeast nitrogen base without amino acids and yeast culture grade agar were purchased from Sunrise Science Products (San Diego, CA). The primers were purchased from Integrated DNA Technologies (Coralville, IA). Mouse monoclonal antibody against HA (purified HA.11 clone 16B12) was purchased from Covance Inc. (Princeton, NJ). Rabbit polyclonal antibodies against HA (ab9110) and Hsp70 (ab79852) were purchased from Abcam (Cambridge, MA). Rabbit polyclonal antibodies against hexokinase were purchased from Rockland Antibodies and Assays (Limerick, PA). Rabbit polyclonal antibody against CBP, the full-length *TbATPa* cDNA clone (GenBank accession no. AAA97428) with yeast optimized codons corresponding to the *T. brucei* ATPase 6, and the full-length *HsMCU* cDNA clone corresponding to the *Homo sapiens* MCU variant 1 (GenBank accession no. NM_138357), as described previously (9), were purchased from GenScript (Piscataway, NJ). Mouse monoclonal antibody against c-MYC was purchased from Santa Cruz Biotechnology, Inc. (Dallas, TX). Rabbit polyclonal antibody against TbCyt c1 was a gift from Dr. Steve Hajduk (University of Georgia, GA). IgG-Sepharose 6 Fast Flow beads were from Pharmacia (GE Healthcare, New York, NY). Rabbit polyclonal antibody against VP16, rabbit polyclonal antibody against V5, rabbit polyclonal antibody against HsMCU, mouse monoclonal antibody against tubulin, and all other reagents of analytical grade were from Sigma (St. Louis, MO). Production of anti-TbMCU antibodies was described before (10). Calmodulin resin was from Stratagene (Agilent Technologies, Santa Clara, CA). The primers were purchased from Integrated DNA Technologies (Coralville, IA).

**HA-tagged TbATPc1, TbATPc2 and TbATPc3 cell lines.** The open reading frames (ORF) of TbATPc1 (354 bp), TbATPc2 (354 bp) or TbATPc3 (351 bp) (TriTrypDB gene ID numbers Tb427tmp.02.2950, Tb427.10.1570, and Tb427.07.1470, respectively) with 6 additional nucleotides prior to the start codons were amplified from *T. brucei* genomic DNA by PCR using the corresponding forward and reverse primers listed in Data Set S2A, which introduced the restriction endonuclease HindII and XbaI sites, respectively. The PCR products were digested with HindIII and XbaI, and cloned in frame into the enzyme-cut pLEW100HA-BSD vector (4) to generate pLEW100HA-BSD(*TbATPc1*), pLEW100100HA-BSD(*TbATPc2*) and pLEW100HA-BSD(*TbATPc3*) (Data Set S2B), respectively. The recombinant plasmids were confirmed by sequencing. The correct constructs pLEW100HA-BSD(*TbATPc1*)*,* pLEW100HA-BSD(*TbATPc2*) and pLEW100HA-BSD(*TbATPc3*) with inducible T7 RNA polymerase-based protein expression system were linearized by NotI and transfected into *T. brucei* PCF 29-13 trypanosomes.

**Mass spectrometry (MS) analysis.** MS analysis of the proteins purified by TAP or HA-tag IP was carried by the Protein Science Facility in the Biotechnology Center at the University of Illinois at Urbana-Champaign. Protein samples (10 μg) were digested with trypsin for 15 min at 55°C using a CEM Liberty microwave digester (11) (CEM Corporation). The digested products were analyzed on a Thermo Scientific Velos Pro mass spectrometer with a Dionex RSLCnano Ultimate 3000 UPLC front end using an Acclaim PepMap RSLC (75-micron X 15 cm, C18 2 micron 100 Angstrom) column (Thermo Scientific) with a linear gradient of 1 to 60 % acetonitrile in 0.1 % formic acid over 120 minutes. The mass spec results were analyzed using the Mascot database search engine (Matrix Science, London) and searched against *T. brucei* 427 TriTrypDB using BLASTp at the website (<http://tritrypdb.org/tritrypdb/>).

**Cell Transfection.** Mid-log phase PCF were transfected with NotI-linearized plasmid DNA or purified PCR products (10 μg) using a Bio-Rad Gene Pulser electroporator as described previously (4). The stable transformants were obtained in SDM-79 medium supplemented with 15% FBS plus appropriate antibiotics (5 μg/ml phleomycin, 50 μg/ml hygromycin, 2 μg/ml puromycin, 10 μg/ml blasticidin, and 15 μg/ml G418).

**Bioinformatics.** Proteins were checked for the presence of mitochondrial targeting sequences (MTS) with Mitoprot (12). Transmembrane domains and coiled–coil (CC) domains were identified with TMHMM (13), and ExPASy Coils Server (<https://embnet.vital-it.ch/software/COILS_form.html>), respectively. Topology of membrane proteins was predicted with Protter (14). Amino acid substitutions were designed according to NCBI Amino Acid Explorer (<https://www.ncbi.nlm.nih.gov/Class/Structure/aa/aa_explorer.cgi>). Protein sequences were aligned with MUSCLE (https://www.ebi.ac.uk/Tools/msa/muscle) and the webserver site (<http://www.bioinformatics.org/sms/index.html>).

**Generation of epitope and “Spaghetti monster” fluorescent proteins (smFPs) tagging constructs or cassettes.** The plasmid pMOTag5H (Addgene #26293) was used as backbone to construct “Spaghetti monster” fluorescent protein (smFP) with epitope tag (c-MYC) plasmid (7). The plasmid was digested with XhoI and SalI to remove the sequence encoding 3xHA tag. smMYC (1118bp, encoding a protein of 40 kDa) was PCR amplified from pCAG_smFP MYC, using the primers smFP-FF and smMYC-FR in Data Set S2A and cloned between the XhoI and SalI sites of the pMOTag vectors using the In-Fusion Cloning Kit (Clontech) to generate pMOTag5mM in Data Set S2B. The correct orientation and sequence of smMYC-epitope in the pMOTag5mM were confirmed by sequencing.

The one-step epitope-tagging protocol reported by Oberholzer et al. (5) was used to produce C-terminal HA-, smMYC- or smV5-tagging cassettes of TbATPβ, TbATPp18, TbANT, TbPiC, and TbATPc1 (TriTrypDB gene ID numbers Tb427.03.1380, Tb427.05.1710, Tb427.10.14820, Tb427tmp.211.1750, and Tb427tmp.02.2950, respectively) for transfection of *T. brucei* PCF WT 427, the triple-smFP-tagged TbMCUC PCF cell line (4, 5), or the TAP-tagged TbMCU PCF29-13 cell line as described above. In brief, the PCR forward and reverse primers included terminal 100-120 nucleotides of each ORF before its stop codon and the reverse complement of the first 100-120 nucleotides of the 3’UTR, respectively, followed in frame by the 21-26 nucleotides of the backbone sequences of pMOTag or pMOTag-smFP vector series (4). The HA-, smMYC- or smV5-epitope tagging cassettes containing an antibiotic resistant gene as a selection marker (hygromycin, phleomycin or puromycin) were generated for cell transfection by PCR using pMOTag4H (5), pMOTag5mM or pMOTag2mV (4) (Data Set S2B and S2C), as template with the corresponding PCR primers of the gene (Data Set S2A).

**Blue-Native PAGE (BN-PAGE) and immunodetection.** Crude *T. brucei* mitochondrial vesicles were isolated from the TAP-tagged TbMCU or TAP-tagged TbMCU plus smV5-tagged TbATPc1 cell line by hypotonic lysis, homogenization and centrifugation, and then frozen in 1 x STE buffer containing 50% glycerol at -80°C for subsequent BN-PAGE analysis. The frozen mitochondrial vesicles were thawed on ice, washed with 1 x STE buffer, lysed in 1 x STE buffer containing 750 mM amino-n-caproic acid (ACA) and 2% dodecylmaltoside (DDM), and cleared by centrifugation as described (Huang and Docampo, 2018). The proteins (100 μg each) of the cleared lysates were loaded onto 4-16% NativePAGE Novex^®^ Bis-Tris Gels, electrophoresed using the Invitrogen NativePAGE^®^ Novex Bis-Tris Gel System, transferred to Immobilon-P PVDF 0.45 μm membrane (Millipore), and then probed with anti-CBP or anti-V5 antibodies as described previously (4).

**Split-ubiquitin membrane-based yeast-two-hybrid (MYTH) assays**

**Yeast strains and media.** *Saccharomyces cerevisiae* NMY51 MYTH reporter strain (MATa his3Δ200 trp1-901 leu2-3, 112 ade2 LYS2::(lexAop)_4_-HIS3 ura3::(lexAop)_8_-lacZ ade2::(lexAop)_8_-ADE2 GAL4) was obtained from Creative Biolabs (NY, USA) and grown on YPDA (46, 78) or Synthetic Defined dropout (SD-DO) media (4), supplemented with or without 1 mM 3-amino-1,2,4-trizole (3-AT), a histidine analog and competitive inhibitor of the *His3* gene product.

**MYTH bait and prey constructs.** The full-length or mutated cDNAs of *TbMCU*, *TbMCUb*, *TbMCUc*, *TbMCUd, TcMCU* (TriTrypDB gene ID number TcCLB.503893.120) and *HsMCU* (GenBank accession no. NM_138357) without N-terminal nucleotide sequences encoding the putative MTS were amplified from *T. brucei* genomic DNA, *T. cruzi* genomic DNA, or the *HsMCU* full-length cDNA clone (synthesized by GenScript) by PCR using the corresponding specific forward and reverse primers (Data Set S2A), which were introduced Sfi I sites, digested with Sfi I at 50^o^C overnight and then cloned in frame into Sfi I-digested MYTH bait (pBT3-SUC) expression vector (8) to generate a set of MYTH bait constructs (Data Set S2B as described in Figs. 3A, 4A, 5A, and 8A. Similar, the full-length or mutated cDNAs of *TbATPa* (GenBank accession no. AAA97428), *TbATPp18* (TriTrypDB gene ID number Tb427.05.1710), *TbATPTb1* (Tb427.10.520), *TbATPTb2* (Tb427.05.2930), *TbATPc* (Tb427tmp.02.2950), *TbATPα* (Tb427.07.7420)*, TbATPβ* (Tb427.03.1380), *TbATPap1* (Tb427.04.3450), *TbATPap2* (Tb427.07.840)*, TbATPap3* (Tb427tmp.02.4120) and *HsATPc* (GenBank accession no. CAG38480.1), without N-terminal nucleotide sequences encoding the putative MTS were amplified from the *TbATPa* cDNA clone (synthesized by Genscript), *T. brucei* genomic DNA, or the *HsATPc* full-length cDNA (generated by fusion PCR using the long primers HsATPc-F and HsATPc-R as listed in Data Set S2A) by PCR using the corresponding specific forward and reverse primers (Data Set S2A, and then cloned in frame into Sfi I-digested MYTH prey expression vectors (pPR3N or pPR3C) (8) to generate a set of MYTH prey constructs (Data Set S2B) as described in Figs. 2A, 3A, 4A, 5A, and 8A.

The double-stranded sequences of the cloned cDNA inserts that express proteins of MCU subunits C-terminally fused to the Cub-LexA-VP16 in pBT3-SUC or ATP synthase subunits N-terminally fused to the NubG-HA in pPR3N or pPR3C were confirmed by sequencing as indicated above. The mutated amino acid residues or artificial WALP (GWWLALALALALALALWWA) sequence(s) were introduced into or replaced the TM domains of MCU subunits in the MYTH bait or prey expression vectors (as described above) by fusion PCR (15) using Phusion Site-Mutagenesis Kit, according to the manufacturer’s instructions.

**MYTH assays of interaction between baits and preys.** The recombinant MYTH bait and prey plasmids harboring full-length, truncated or mutated MCU or ATP synthase subunits were co-transformed into the yeast NMY51 strain by LiOAc-mediated transformation as described (16), and cultured successively on the dual, triple and quadruple SD media (SD/-Leu-Trp, SD/-Leu-Trp-His, SD/-Leu-Trp-His-Ade; shorted as SD-2DO, SD-3DO, SD-4DO). After incubation at 30^o^C for 3-4 days, colonies grown on the selective SD plates were further screened by cultivating on SD-4DO/X-gal media, and β-galactosidase (β-Gal) activity was measured using Yeast β-Galactosidase Assay Kit as described previously (4) to test the expression of the reporter gene *lacZ*. The β-Gal assay was repeated 3 times for a number of colonies (as indicated), followed by calculation of standard deviations. Statistical significance was calculated using the Student’s *t* test. MYTH colonies were analyzed by western blotting, immunofluorescence microscopy, and co-immunoprecipitation as described below.

**Immunofluorescence microscopy.** To determine the localization and expression of TbATPβ, TbATPp18, TbANT, TbPiC TbATPc1/c2/c3 subunits and TbMCUC subunits in *T. brucei*, trypanosome live cells were labeled with Mitotracker Red CMXRos (Invitrogen), fixed with 4% paraformaldehyde, incubated with antibodies against HA, c-MYC, V5, CBP or TbMCU and then stained with Alexa Fluor-conjugate secondary antibodies (Data Set S2D) as described previously (4). Immunofluorescence of yeast was also performed as described previously (4). After labeled with primary and secondary antibodies, the trypanosome or yeast cells on the coverslips were counterstained with 4′,6-diamidino-2-phenylindole (DAPI) before mounting with Gold ProLong Gold antifade reagent (Molecular Probes). Differential interference contrast and fluorescent optical images were captured using an Olympus IX-71 inverted fluorescence microscope with a Photometrix CoolSnap^HQ^ charge-coupled device camera driven by DeltaVision software (Applied Precision, Seattle, WA). Images were deconvolved for 15 cycles using Softwarx deconvolution software. Pearson’s correlation coefficients (PCC) were calculated using the Softwarx software by measuring the whole-cell images.

**Immunoprecipitation studies.** *T. brucei* MCU was immunoprecipitated from *in situ* HA-tagged TbATPβ, TbATPp18, TbANT, TbPiC PCF427 cell lines, or HA-tagged TbATPc1/c2/c3 overexpressing PCF 29-13 cell lines. *T. brucei* MCU complex and the ATP synthase (subunits c, β and p18) were co-immunoprecipitated from the quadruple *in situ* smFP-tagged (triple-smFP-tagged TbMCUC plus smMYC-tagged TbATPc, TbATPβ or TbATPp18) cell line or the TAP-tagged TbMCU plus smV5-tagged TbATPc, TbATPβ or TbATPp18 cell line as described above. Human MCU and the ATPc were co-immunoprecipitated from HEK-293T, the MCU-KO (as a negative control), or HeLa cells. The IPs and Co-IPs were carried out under native conditions using antibodies against HA, MYC, CBP, V5, HsMCU or HsATPc with Pierce Classic Protein G IP kit or HA-Tag IP/Co-IP kit according to the manufacturer’s instructions as described (4). After IP/Co-IP, 10 µl of each eluate (or immunoprecipitate) and 30 µl of cleared lysate were loaded on 10-12% SDS-PAGE gels for western blot analyses with specific antibodies (Data Set S2D) using anti-TbCyt *c*_1_ or anti-HsHsp70 antibody for controls. Yeast IP/Co-IP was performed under native conditions with antibodies against HA and VP16 (Data Set S2D) as described previously (4), using anti-HK antibody for controls.

**Western blot analyses.** *T. brucei*, human (HEK and HeLa), and zymolyase-digested yeast NMY51 cells were lysed with RIPA buffer containing protease inhibitor tablet as described previously (4). Total cell lysates, mitochondrial vesicle lysates, or immunoprecipitation eluates were mixed with 2 × Laemmli sample buffer (BioRad) at 1:1 ratio (volume/volume), directly loaded (for *T. brucei* and human proteins) or loaded after boiling for 5 minutes (for yeast proteins). The separated proteins were transferred onto nitrocellulose membranes or Immobilon-P PVDF 0.45 μm membranes using a Bio-Rad transblot apparatus. The membranes were incubated with antibodies against CBP, MYC, TbMCU, HA, V5, hexokinase, HsMCU, HsATPc, HsHsp70 or tubulin and then with HPR-conjugate secondary antibodies (Data Set S2D) as described previously (4). After washing, the immunoblots were visualized using Pierce ECL Western blotting substrate according to the manufacturer’s instructions.

**Adenine nucleotide levels.** *TbMCUb,* *TbMCUc* and *TbMCUd* RNAi PCF trypanosomes, which we previously generated (4), were cultivated in SDM-79 for 4 days with or without tetracycline or in a glucose-depleted medium (SDM-80) containing 5.2 mM L-proline for 2 days in the absence or presence of tetracycline after a 2-day culture in SDM-79 with or without tetracycline. Adenine nucleotides (AMP, ADP and ATP) were extracted from the cells using perchloric acid, neutralized, and then quantified using an ATP Determination Kit (Invitrogen) with adenylate kinase and/or nucleoside-diphosphate kinase (NDK; Sigma) as described previously (10).

**Statistical analyses.** All values are expressed as means ± s.d.. Significant differences between treatments were compared using unpaired Student’s t-test. Differences were considered statistically significant at P < 0.05, and n refers to the number of experiments performed. All statistical analyses were conducted using GraphPad Prism 5 (GraphPad Software, San Diego, CA).

**References**

1. Wirtz E, Leal S, Ochatt C, Cross GA. 1999. A tightly regulated inducible expression system for conditional gene knock-outs and dominant-negative genetics in Trypanosoma brucei. Molecular and biochemical parasitology 99:89-101.

2. Cunningham I, Honigberg BM. 1977. Infectivity reacquisition by Trypanosoma brucei brucei cultivated with tsetse salivary glands. Science 197:1279-82.

3. Huang G, Bartlett PJ, Thomas AP, Moreno SN, Docampo R. 2013. Acidocalcisomes of Trypanosoma brucei have an inositol 1,4,5-trisphosphate receptor that is required for growth and infectivity. Proceedings of the National Academy of Sciences of the United States of America 110:1887-92.

4. Huang G, Docampo R. 2018. The Mitochondrial Ca(2+) Uniporter Complex (MCUC) of Trypanosoma brucei Is a Hetero-oligomer That Contains Novel Subunits Essential for Ca(2+) Uptake. MBio 9.

5. Oberholzer M, Morand S, Kunz S, Seebeck T. 2006. A vector series for rapid PCR-mediated C-terminal in situ tagging of Trypanosoma brucei genes. Molecular and biochemical parasitology 145:117-20.

6. Carnes J, Schnaufer A, McDermott SM, Domingo G, Proff R, Steinberg AG, Kurtz I, Stuart K. 2012. Mutational analysis of Trypanosoma brucei editosome proteins KREPB4 and KREPB5 reveals domains critical for function. RNA 18:1897-909.

7. Viswanathan S, Williams ME, Bloss EB, Stasevich TJ, Speer CM, Nern A, Pfeiffer BD, Hooks BM, Li WP, English BP, Tian T, Henry GL, Macklin JJ, Patel R, Gerfen CR, Zhuang X, Wang Y, Rubin GM, Looger LL. 2015. High-performance probes for light and electron microscopy. Nat Methods 12:568-76.

8. Lentze N, Auerbach D. 2008. Membrane-based yeast two-hybrid system to detect protein interactions. Curr Protoc Protein Sci Chapter 19:Unit 19 17.

9. Chiurillo MA, Lander N, Bertolini MS, Storey M, Vercesi AE, Docampo R. 2017. Different Roles of Mitochondrial Calcium Uniporter Complex Subunits in Growth and Infectivity of Trypanosoma cruzi. MBio 8.

10. Huang G, Vercesi AE, Docampo R. 2013. Essential regulation of cell bioenergetics in Trypanosoma brucei by the mitochondrial calcium uniporter. Nat Commun 4:2865.

11. Sieracki NA, Tian S, Hadt RG, Zhang JL, Woertink JS, Nilges MJ, Sun F, Solomon EI, Lu Y. 2014. Copper-sulfenate complex from oxidation of a cavity mutant of Pseudomonas aeruginosa azurin. Proc Natl Acad Sci U S A 111:924-9.

12. Claros MG. 1995. MitoProt, a Macintosh application for studying mitochondrial proteins. Comput Appl Biosci 11:441-7.

13. Krogh A, Larsson B, von Heijne G, Sonnhammer EL. 2001. Predicting transmembrane protein topology with a hidden Markov model: application to complete genomes. Journal of Moleculr Biology 305:567-580.

14. Omasits U, Ahrens CH, Muller S, Wollscheid B. 2014. Protter: interactive protein feature visualization and integration with experimental proteomic data. Bioinformatics 30:884-6.

15. Ho SN, Hunt HD, Horton RM, Pullen JK, Pease LR. 1989. Site-directed mutagenesis by overlap extension using the polymerase chain reaction. Gene 77:51-9.

16. Gietz RD, Schiestl RH. 2007. High-efficiency yeast transformation using the LiAc/SS carrier DNA/PEG method. Nat Protoc 2:31-4.
