# Supplementary figures and images for: The Mitochondrial Calcium Uniporter Interacts with Subunit c of the ATP Synthase of Trypanosomes and Humans
Source: mBio. 2020 Mar 17;11(2):e00268-20. doi: 10.1128/mBio.00268-20 (PMC7078472; doi:10.1128/mBio.00268-20)

A

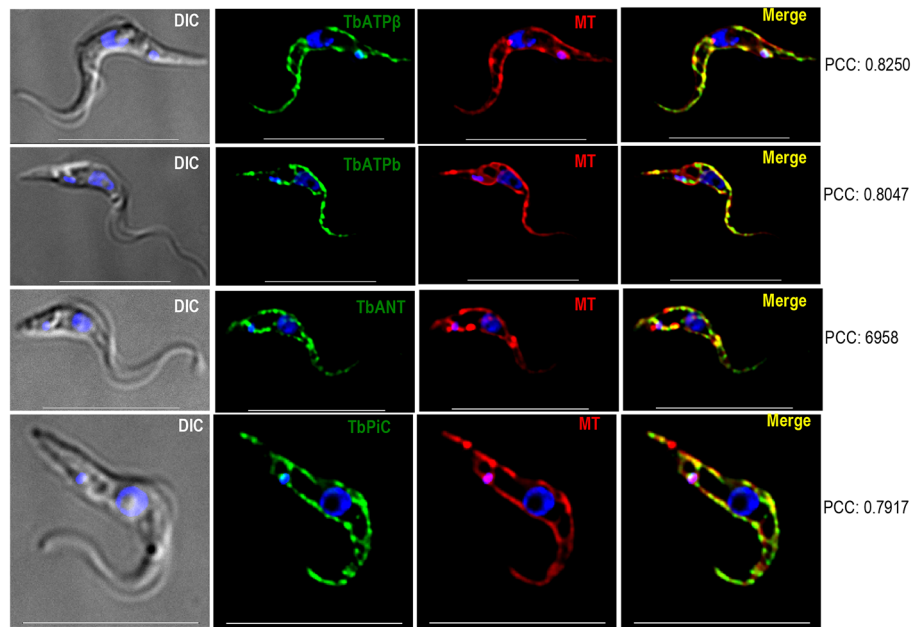

B

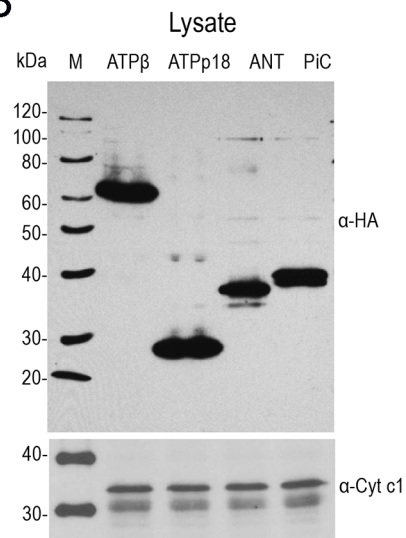

C

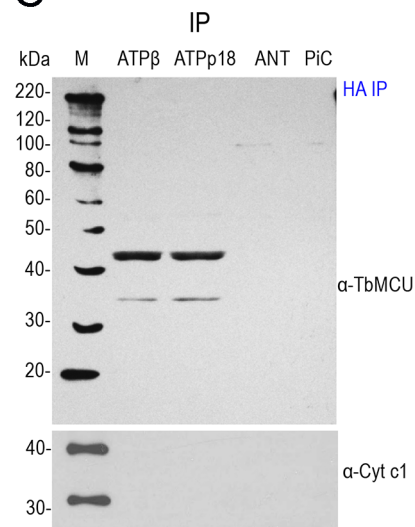

Supplement: FIG S2 [file mBio.00268-20-sf002.pdf]

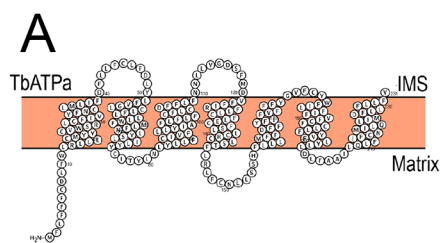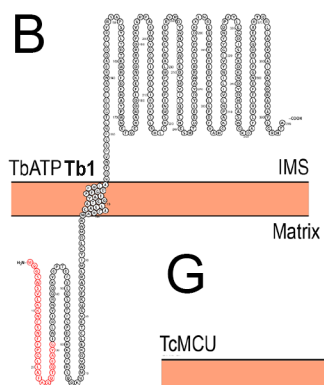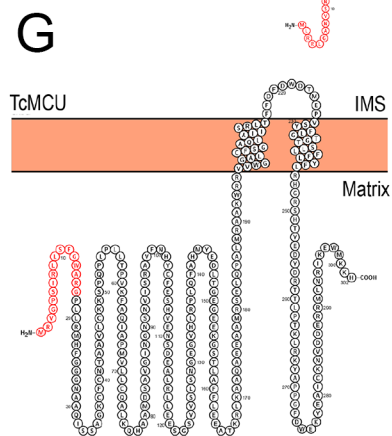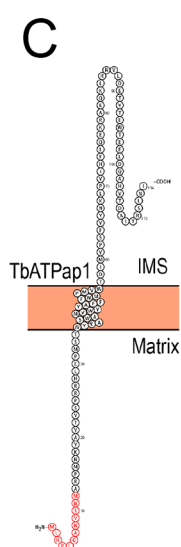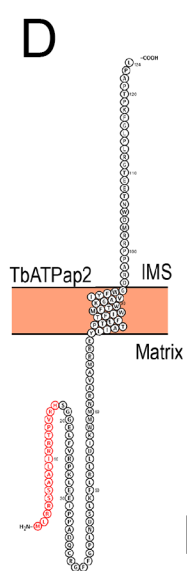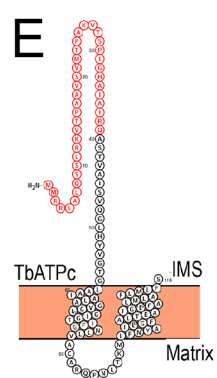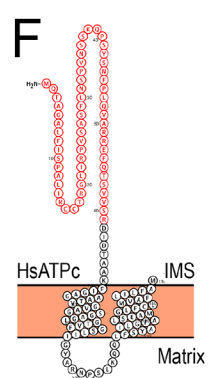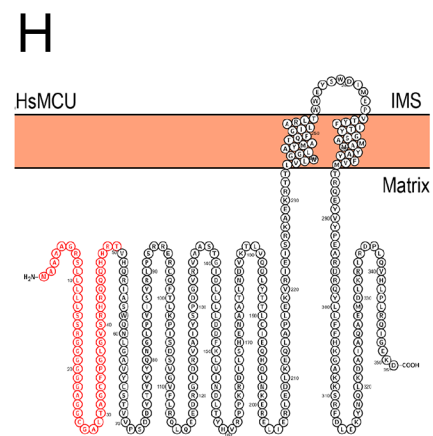

Supplement: FIG S3 [file mBio.00268-20-sf003.pdf]

**A**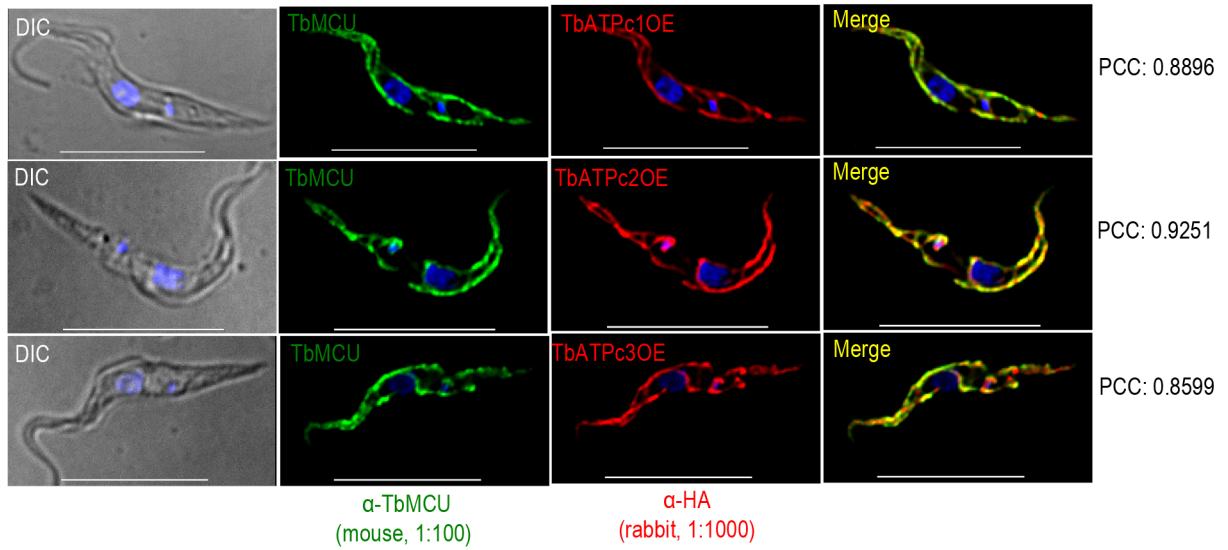**B**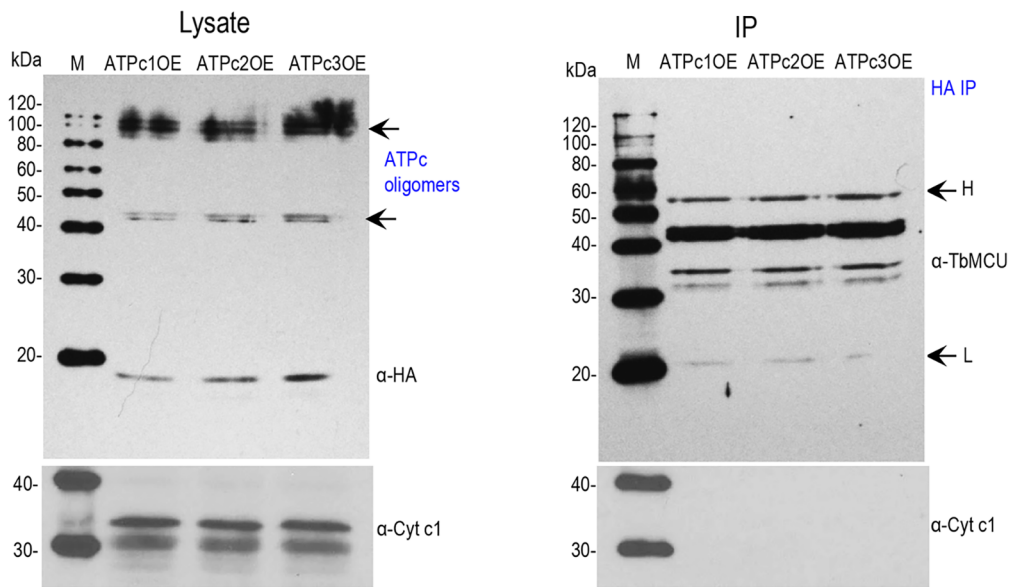

Supplement: FIG S5 [file mBio.00268-20-sf005.pdf]

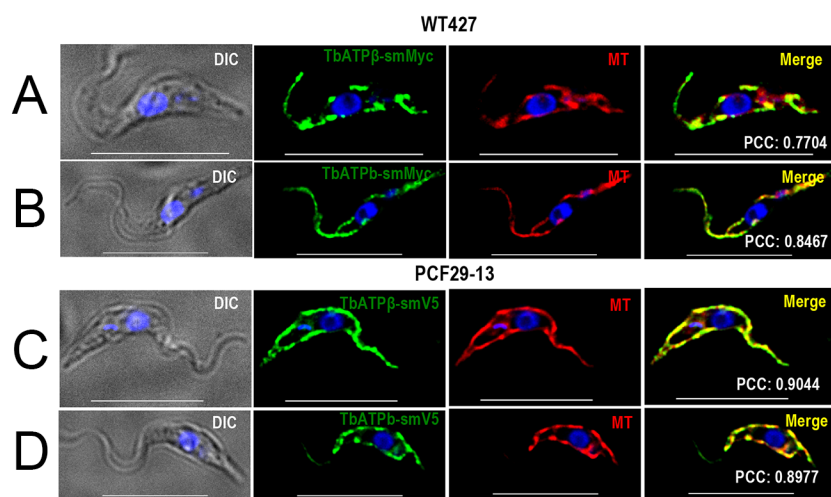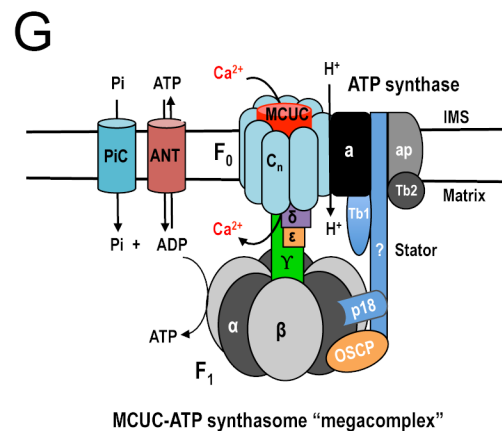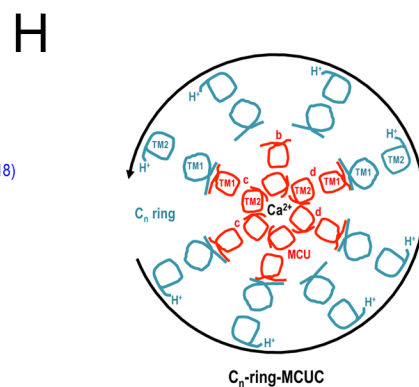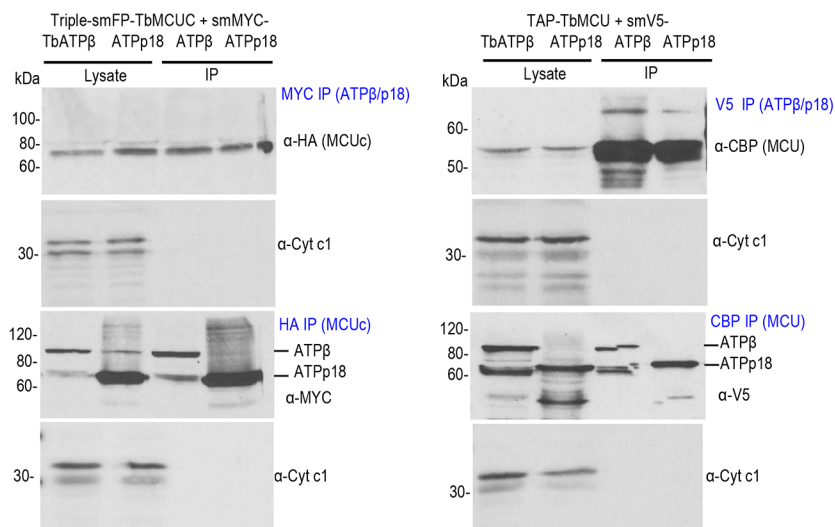

Supplement: FIG S6 [file mBio.00268-20-sf006.pdf]
