# Supplementary material for: The Mitochondrial Calcium Uniporter Interacts with Subunit c of the ATP Synthase of Trypanosomes and Humans
Source: mBio. 2020 Mar 17;11(2):e00268-20. doi: 10.1128/mBio.00268-20 (PMC7078472; doi:10.1128/mBio.00268-20)
Supplement: FIG S4 [file mBio.00268-20-sf004.pdf]

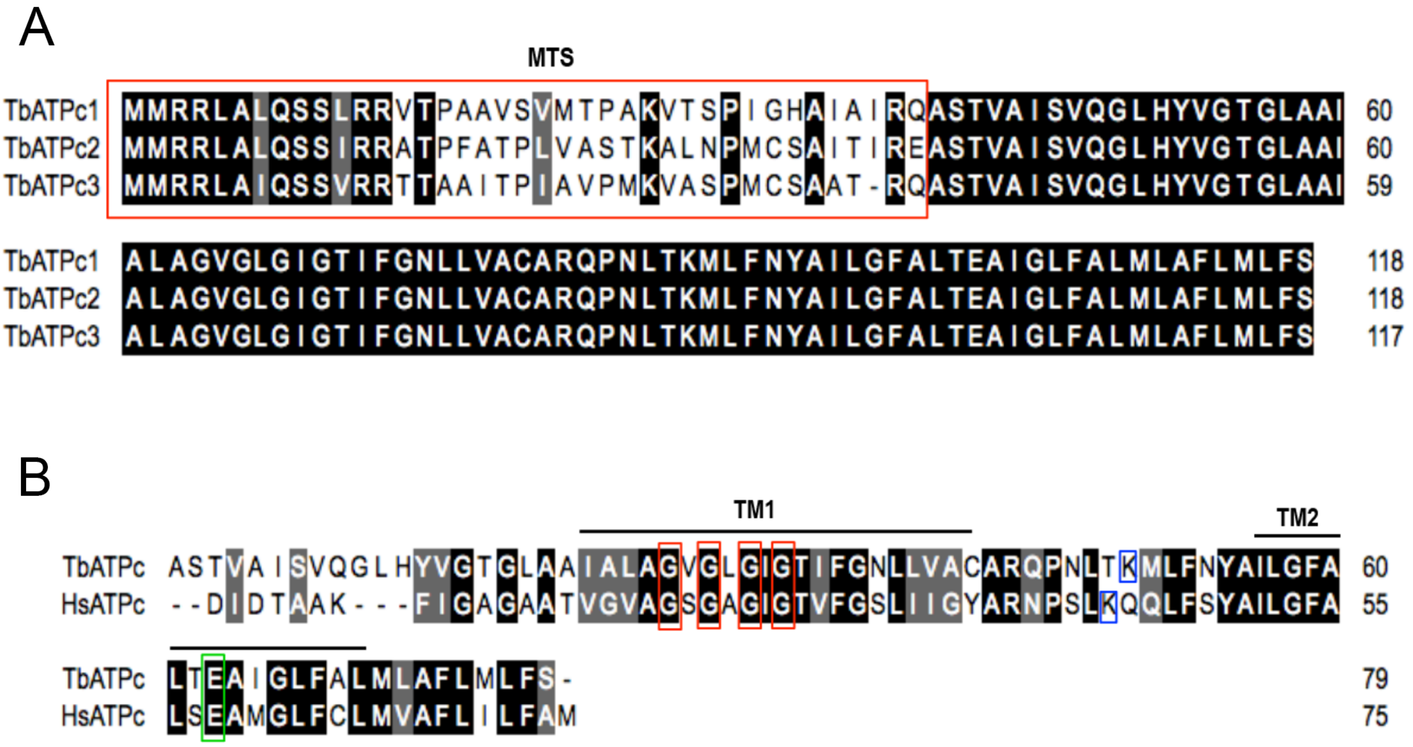
A
MTS
TbATPc1 MMRRLALQSSLRRVTPAAVSVMTPAKVTSPIGHAIAIRQASTVAISVQGLHYVGTGLAAI 60
TbATPc2 MMRRLALQSSIRRATPFATPLVASTKALNPMCSAITIREASTVAISVQGLHYVGTGLAAI 60
TbATPc3 MMRRLAIQSSVRRTTAAITPIAVPMKVASPMCSAAT-RQASTVAISVQGLHYVGTGLAAI 59
TbATPc1 ALAGVGLGIGTIFGNLLVACARQPNLTKMLFNYAILGFALTEAIGLFALMLAFLMLFS 118
TbATPc2 ALAGVGLGIGTIFGNLLVACARQPNLTKMLFNYAILGFALTEAIGLFALMLAFLMLFS 118
TbATPc3 ALAGVGLGIGTIFGNLLVACARQPNLTKMLFNYAILGFALTEAIGLFALMLAFLMLFS 117
B
TM1
TM2
TbATPc ASTVAISVQGLHYVGTGLAAIALAGVGLGIGTIFGNLLVACARQPNLTKMLFNYAILGFA 60
HsATPc --DIDTAAK---FIGAGAATVGVAGSGAGIGTVFGSLIIGYARNPSLKQQLFSYAILGFA 55
TbATPc LTEAIGLFALMLAFLMLFS- 79
HsATPc LSEAMGLFCLMVAFLILFAM 75
