# Supplementary material for: The Mitochondrial Calcium Uniporter Interacts with Subunit c of the ATP Synthase of Trypanosomes and Humans
Source: mBio. 2020 Mar 17;11(2):e00268-20. doi: 10.1128/mBio.00268-20 (PMC7078472; doi:10.1128/mBio.00268-20)

Figure S7: Full-size blots

Fig 1C

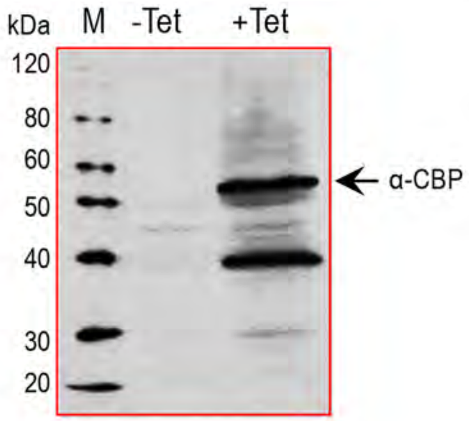

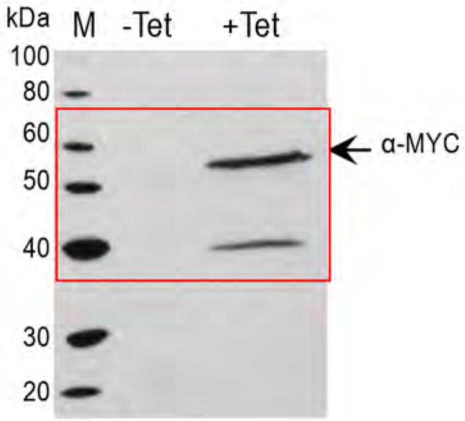

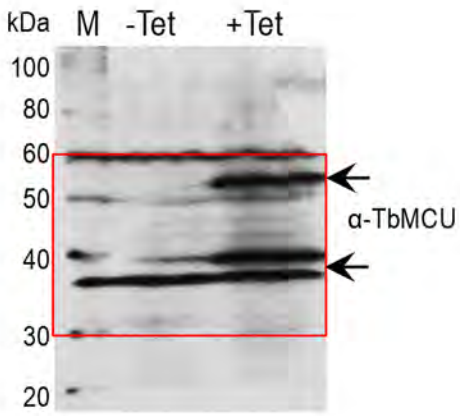

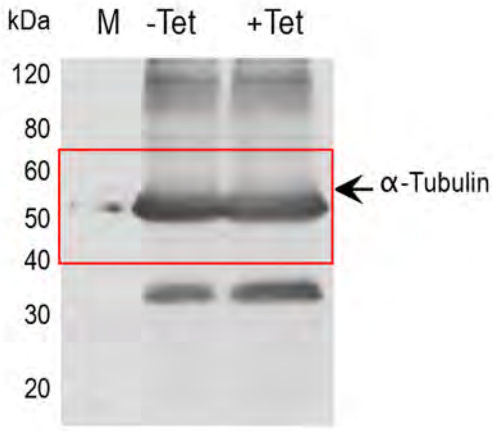

Fig 2A

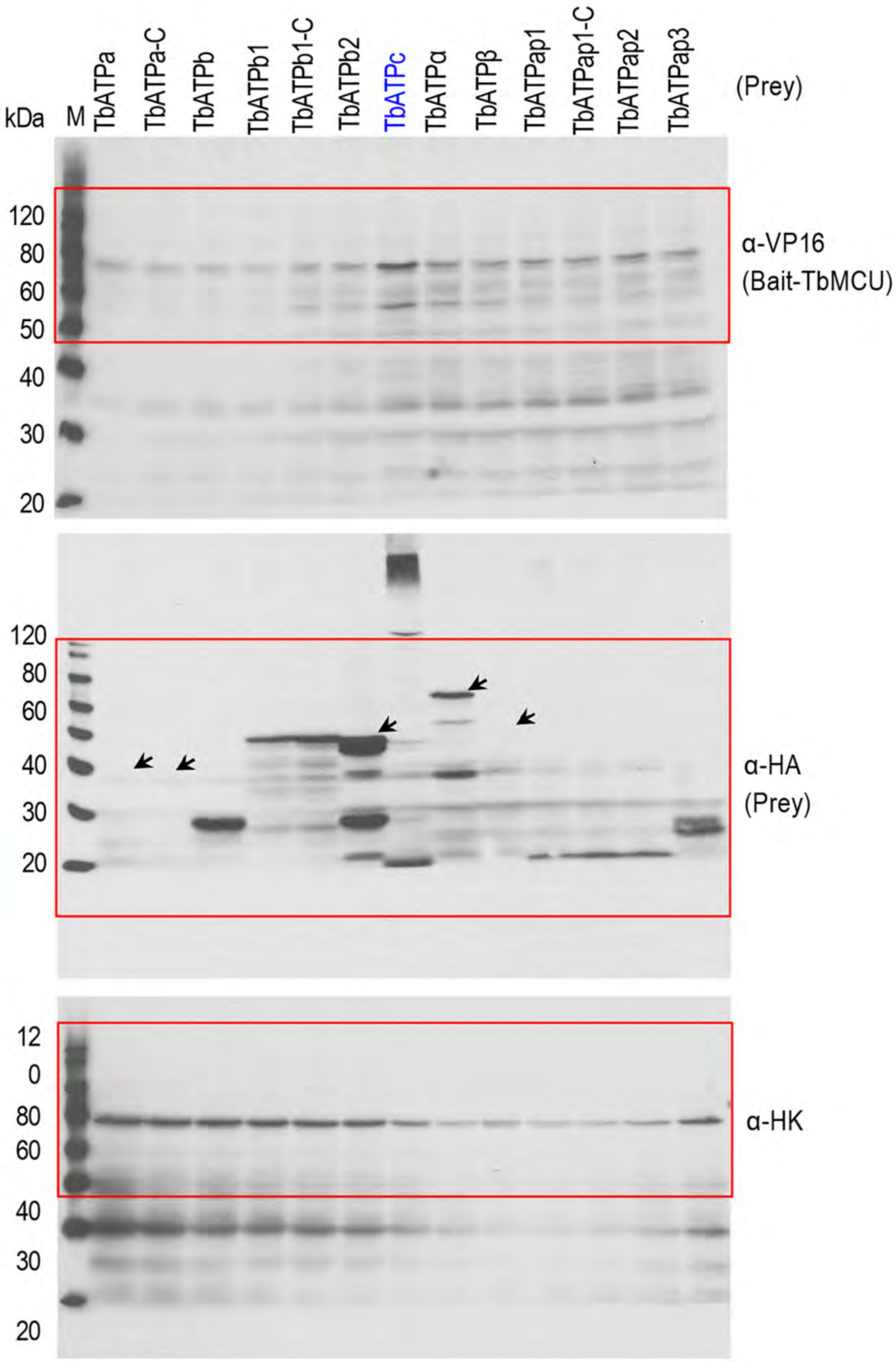

kDa
M
TbATPa
TbATPa-C
TbATPb
TbATPb1
TbATPb1-C
TbATPb2
TbATPc
TbATPα
TbATPβ
TbATPap1
TbATPap1-C
TbATPap2
TbATPap3
(Prey)
120
80
60
50
40
30
20
α-VP16
(Bait-TbMCU)
120
80
60
40
30
20
α-HA
(Prey)
12
0
80
60
40
30
20
α-HK

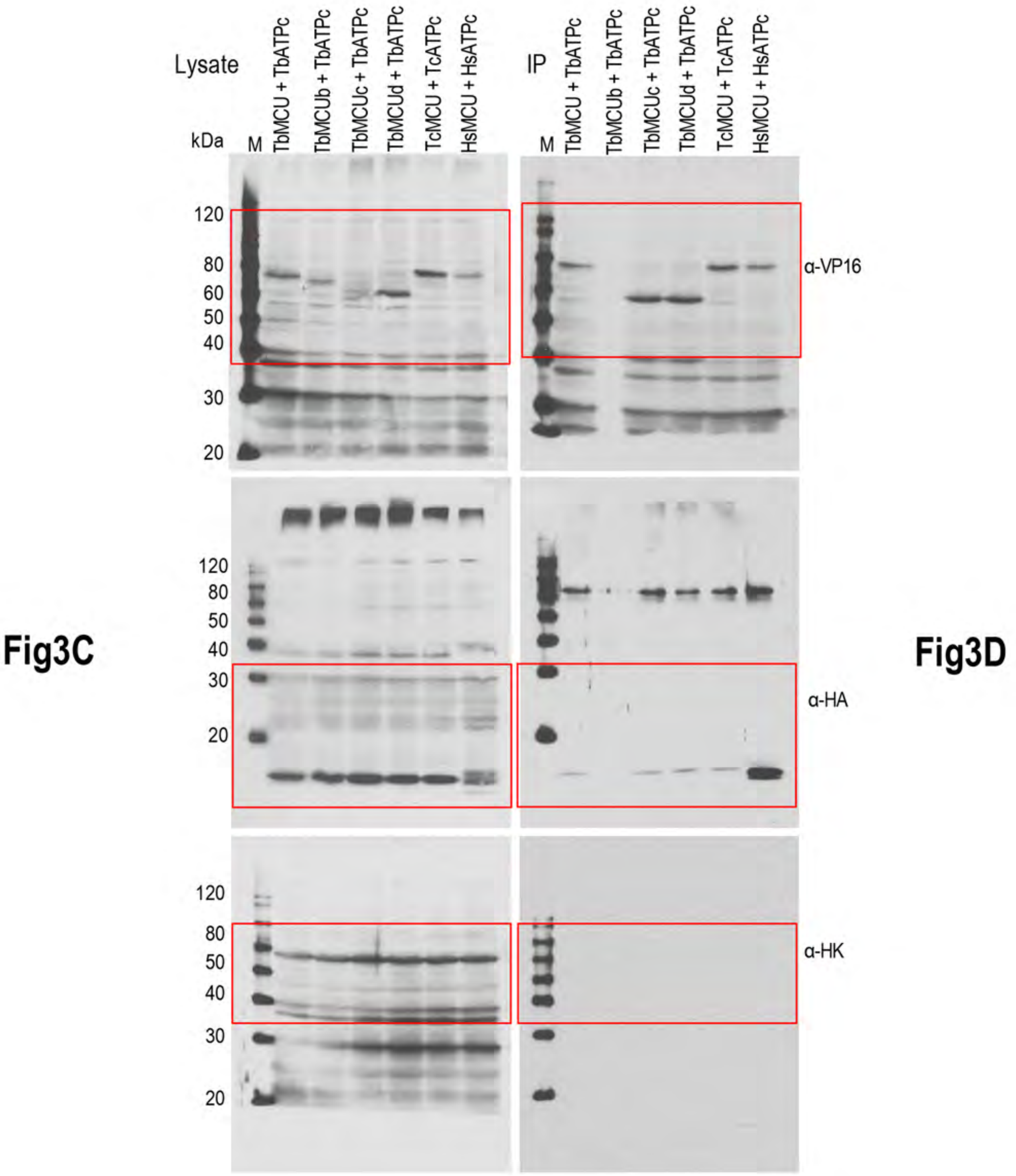

Lysate
IP
kDa
M
TbMCU + TbATPc
TbMCUb + TbATPc
TbMCUc + TbATPc
TbMCUd + TbATPc
TcMCU + TcATPc
HsMCU + HsATPc
120
80
60
50
40
30
20
α-VP16
α-HA
α-HK
Fig3C
Fig3D

Fig 4E

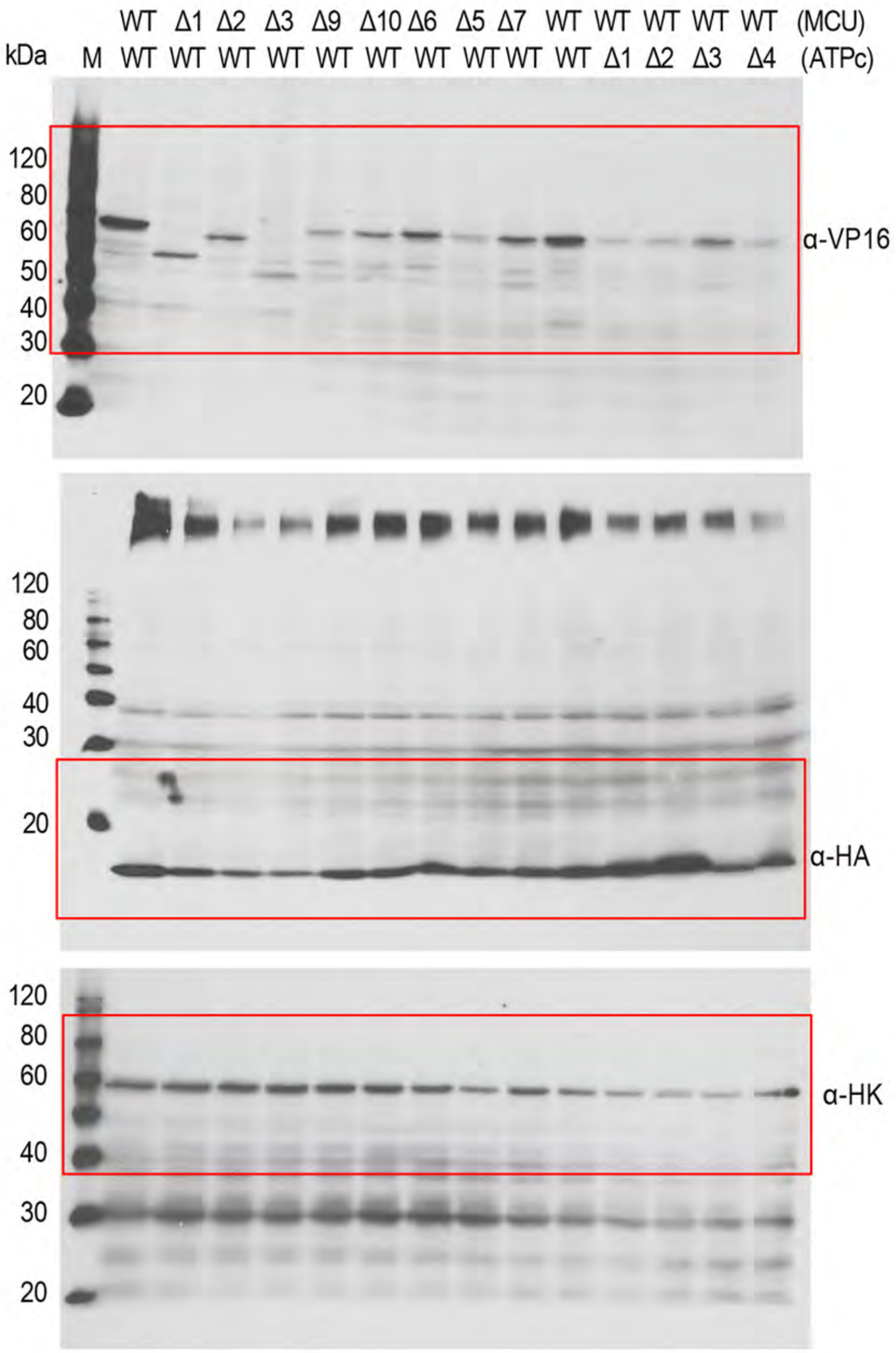

WT Δ1 Δ2 Δ3 Δ9 Δ10 Δ6 Δ5 Δ7 WT WT WT WT WT (MCU)
kDa M WT WT WT WT WT WT WT WT WT WT Δ1 Δ2 Δ3 Δ4 (ATPc)
120
80
60
50
40
30
20
α-VP16
120
80
60
40
30
20
α-HA
120
80
60
40
30
20
α-HK

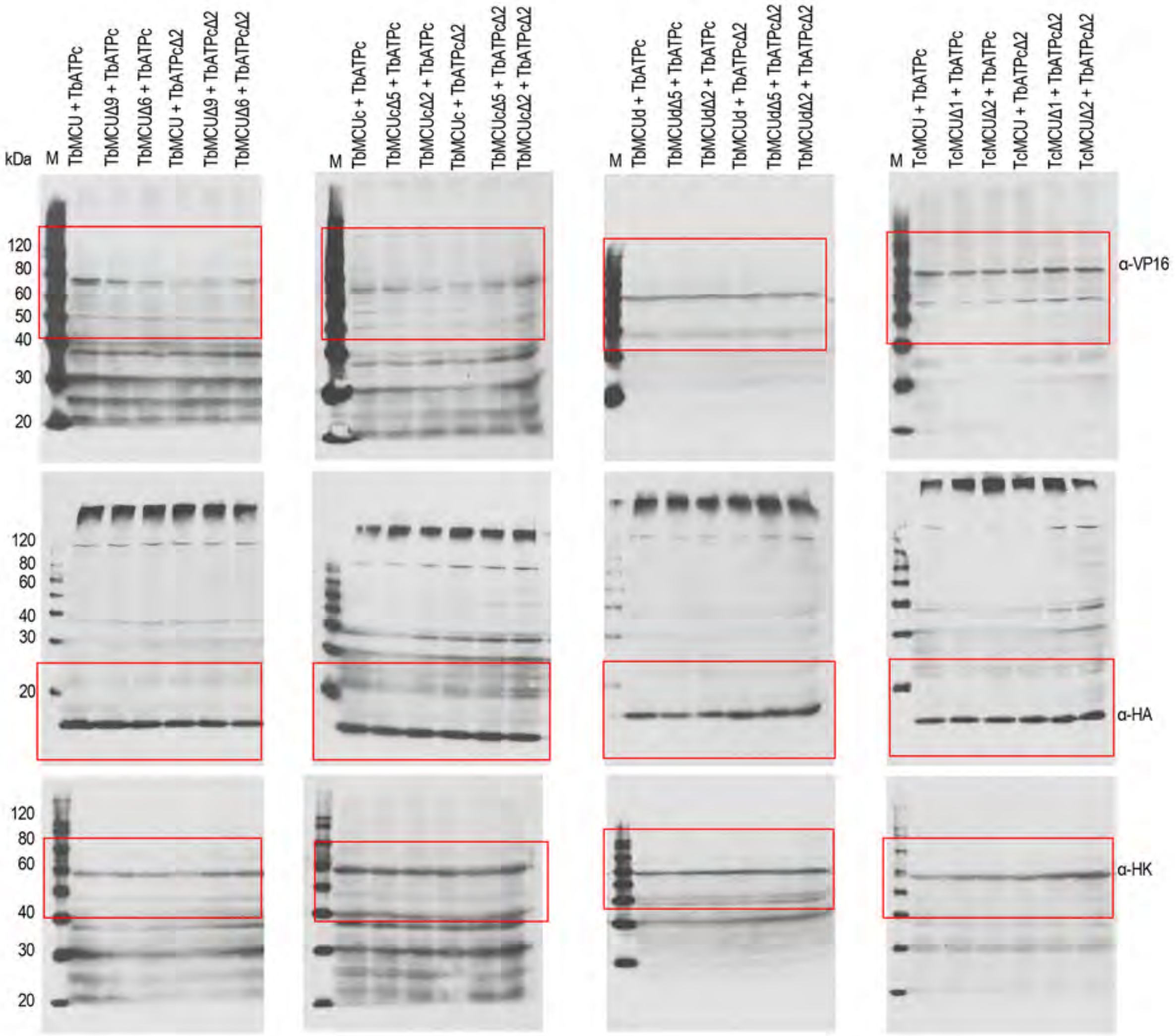
kDa
M
TbMCU + TbATPc
TbMCUΔ9 + TbATPc
TbMCUΔ6 + TbATPc
TbMCU + TbATPcΔ2
TbMCUΔ9 + TbATPcΔ2
TbMCUΔ6 + TbATPcΔ2
TbMCUc + TbATPc
TbMCUcΔ5 + TbATPc
TbMCUcΔ2 + TbATPc
TbMCUc + TbATPcΔ2
TbMCUcΔ5 + TbATPcΔ2
TbMCUcΔ2 + TbATPcΔ2
TbMCUd + TbATPc
TbMCUdΔ5 + TbATPc
TbMCUdΔ2 + TbATPc
TbMCUd + TbATPcΔ2
TbMCUdΔ5 + TbATPcΔ2
TbMCUdΔ2 + TbATPcΔ2
TcMCU + TbATPc
TcMCUΔ1 + TbATPc
TcMCUΔ2 + TbATPc
TcMCU + TbATPcΔ2
TcMCUΔ1 + TbATPcΔ2
TcMCUΔ2 + TbATPcΔ2
120
80
60
50
40
30
20
α-VP16
α-HA
α-HK
Fig 5C

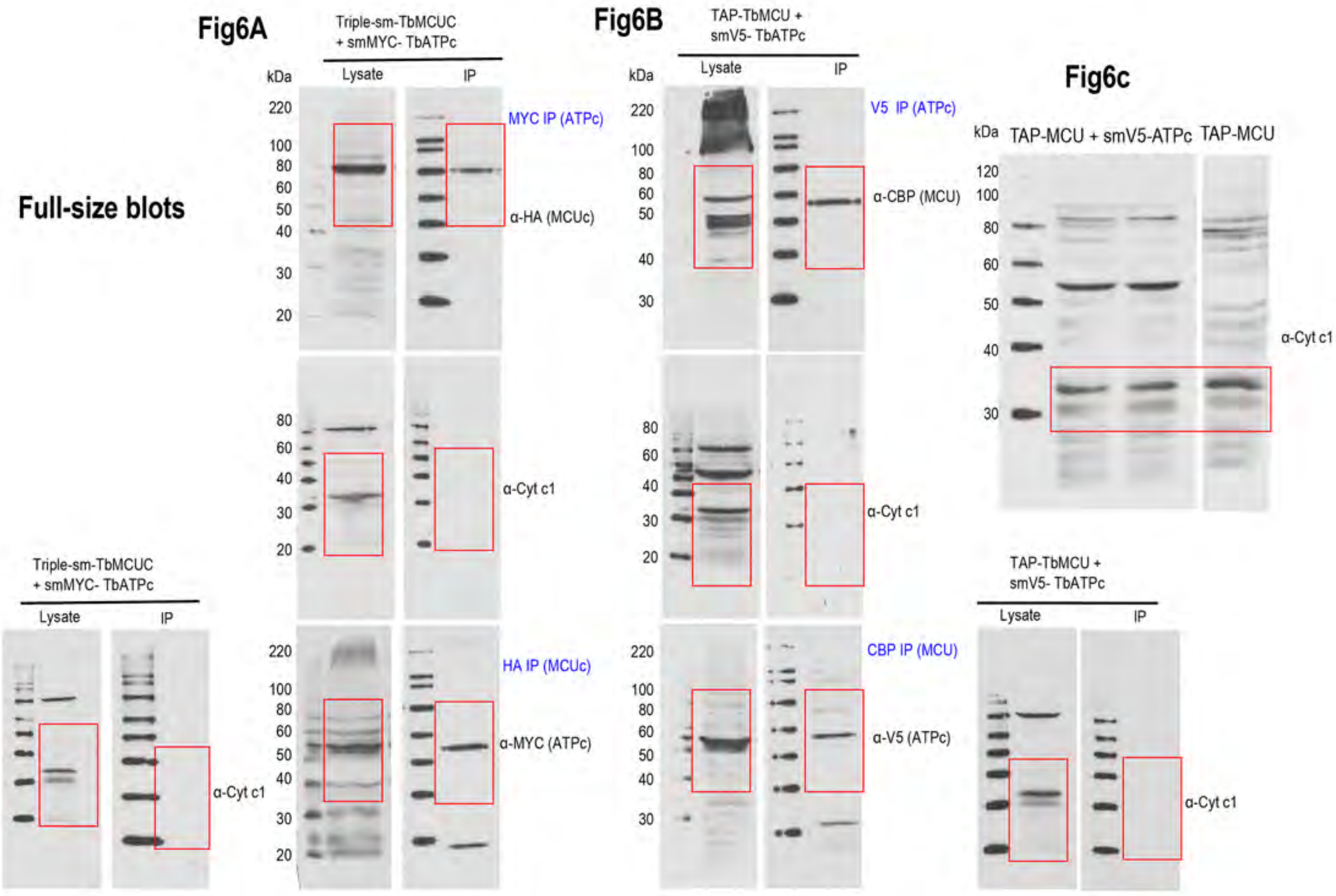
Fig6A
Full-size blots
Triple-sm-TbMCUC
+ smMYC- TbATPc
Lysate
IP
kDa
220
100
80
60
50
40
30
20
MYC IP (ATPc)
α-HA (MCUc)
α-Cyt c1
HA IP (MCUc)
α-MYC (ATPc)
Fig6B
TAP-TbMCU +
smV5- TbATPc
V5 IP (ATPc)
α-CBP (MCU)
CBP IP (MCU)
α-V5 (ATPc)
Fig6c
TAP-MCU + smV5-ATPc
TAP-MCU
120

Full-size blots

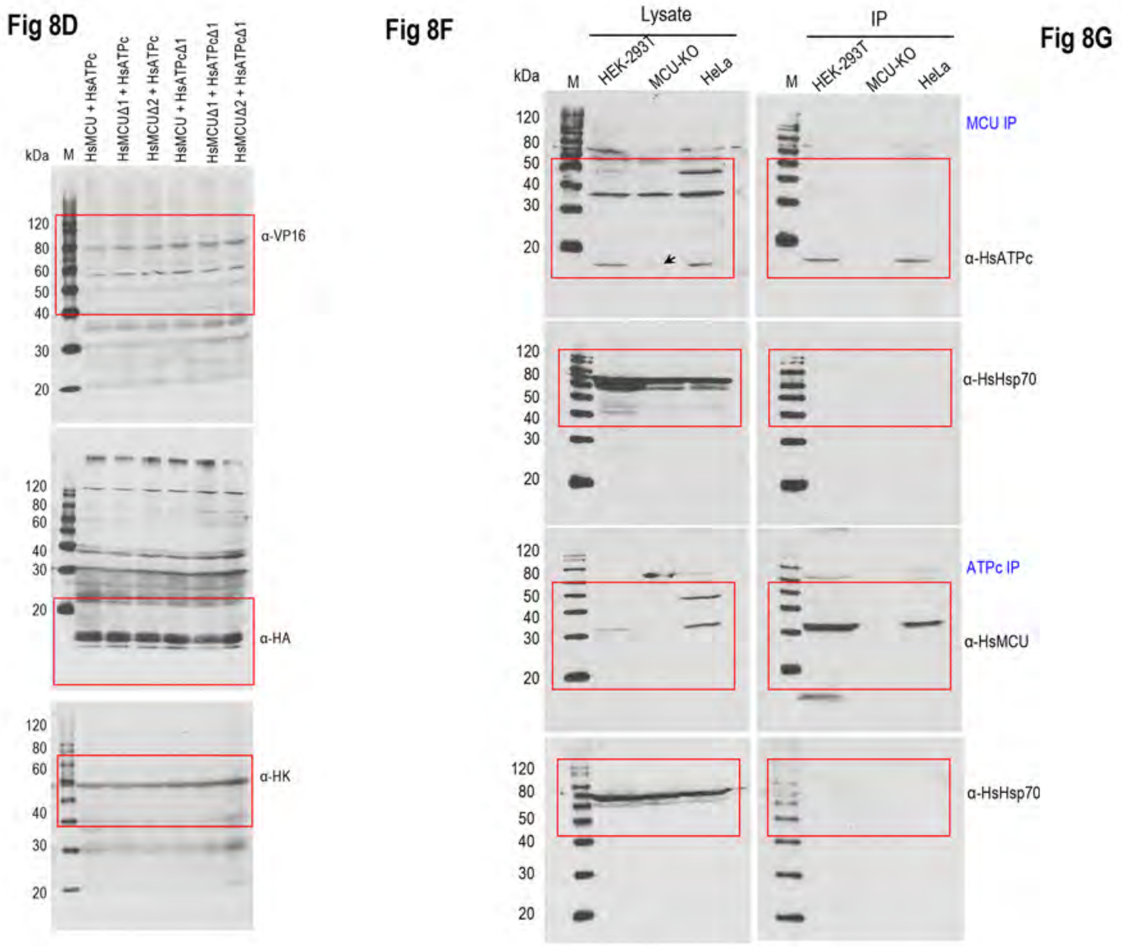

Figure S2: Full blots

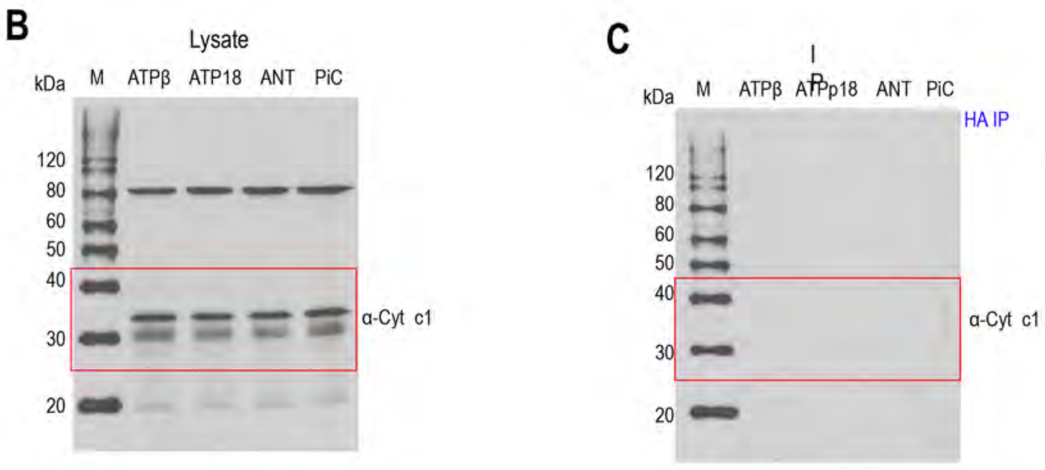

Full blots: Figure S6B

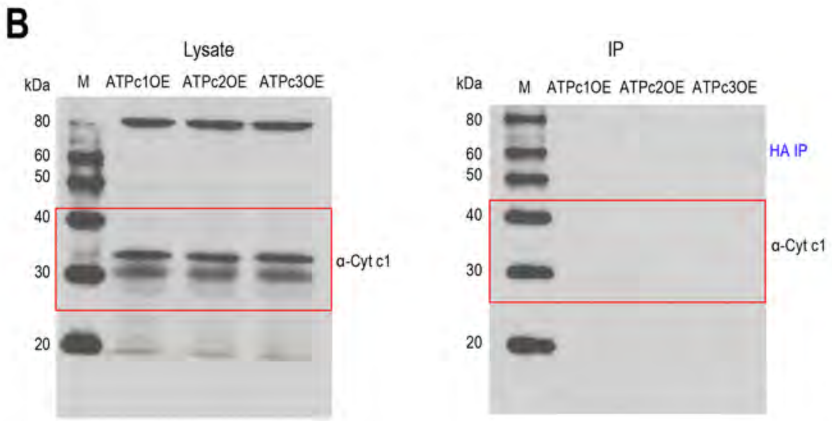

Full-size blots

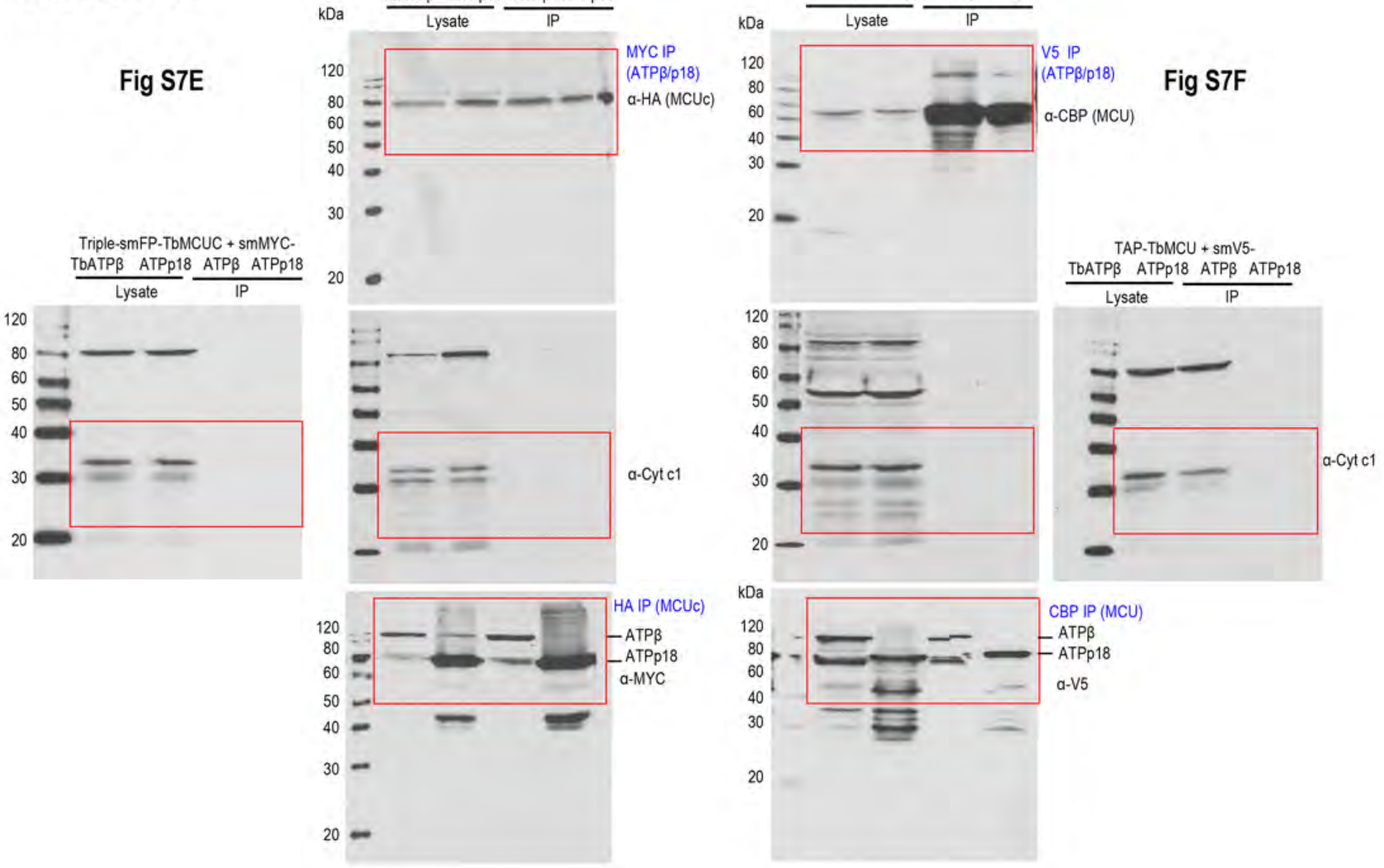

Supplement: FIG S7 [file mBio.00268-20-sf007.pdf]
